# The *Arthrobacter arilaitensis* Re117 Genome Sequence Reveals Its Genetic Adaptation to the Surface of Cheese

**DOI:** 10.1371/journal.pone.0015489

**Published:** 2010-11-24

**Authors:** Christophe Monnet, Valentin Loux, Jean-François Gibrat, Eric Spinnler, Valérie Barbe, Benoit Vacherie, Frederick Gavory, Edith Gourbeyre, Patricia Siguier, Michaël Chandler, Rayda Elleuch, Françoise Irlinger, Tatiana Vallaeys

**Affiliations:** 1 INRA, UMR782 Génie et microbiologie des procédés alimentaires, Thiverval-Grignon, France; 2 AgroParisTech, UMR782 Génie et microbiologie des procédés alimentaires, Thiverval-Grignon, France; 3 INRA, UR1077 Mathématique, Informatique et Génome, Jouy-en-Josas, France; 4 CEA/DSV/IG/Genoscope, Evry, France; 5 UMR5100 Laboratoire de Microbiologie et Génétique Moléculaire, CNRS-Université Paul Sabatier, Toulouse, France; 6 Laboratoire de Génie Enzymatique et de Microbiologie, Université des Sciences de Sfax, Sfax, Tunisia; 7 UMR5119 Ecosystèmes lagunaires, CNRS – CC093 Université Montpellier II, Montpellier, France; University of Hyderabad, India

## Abstract

*Arthrobacter arilaitensis* is one of the major bacterial species found at the surface of cheeses, especially in smear-ripened cheeses, where it contributes to the typical colour, flavour and texture properties of the final product. The *A. arilaitensis* Re117 genome is composed of a 3,859,257 bp chromosome and two plasmids of 50,407 and 8,528 bp. The chromosome shares large regions of synteny with the chromosomes of three environmental *Arthrobacter* strains for which genome sequences are available: *A. aurescens* TC1, *A. chlorophenolicus* A6 and *Arthrobacter* sp. FB24. In contrast however, 4.92% of the *A. arilaitensis* chromosome is composed of ISs elements, a portion that is at least 15 fold higher than for the other *Arthrobacter* strains. Comparative genomic analyses reveal an extensive loss of genes associated with catabolic activities, presumably as a result of adaptation to the properties of the cheese surface habitat. Like the environmental *Arthrobacter* strains, *A. arilaitensis* Re117 is well-equipped with enzymes required for the catabolism of major carbon substrates present at cheese surfaces such as fatty acids, amino acids and lactic acid. However, *A. arilaitensis* has several specificities which seem to be linked to its adaptation to its particular niche. These include the ability to catabolize D-galactonate, a high number of glycine betaine and related osmolyte transporters, two siderophore biosynthesis gene clusters and a high number of Fe^3+^/siderophore transport systems. In model cheese experiments, addition of small amounts of iron strongly stimulated the growth of *A. arilaitensis*, indicating that cheese is a highly iron-restricted medium. We suggest that there is a strong selective pressure at the surface of cheese for strains with efficient iron acquisition and salt-tolerance systems together with abilities to catabolize substrates such as lactic acid, lipids and amino acids.

## Introduction

Smear-ripened cheeses, such as Livarot, Maroilles, Munster, Limburger and Tilsit, are characterized by a complex surface microflora, composed of various species of yeasts and bacteria. During the first hours of the manufacturing procedure, lactic acid bacteria grow in the milk, resulting in a decrease of pH. This acidification, in combination with the effect of rennet, results in the formation of a coagulum. Fresh curd blocks are then shaped from the curd grains, brined, and colonization by acid-tolerant yeasts occurs within a few days. The yeasts increase the pH by assimilating lactate and producing ammonia, thereby favouring the growth of less acid-tolerant ripening bacteria. These bacteria contribute to a large extent to the development of the typical colour, flavour and texture of the smear-ripened cheeses. Their rapid growth also reduces the risk of contamination with pathogens such as *Listeria monocytogenes*. In many cases, the dominant bacteria of smear-ripened cheeses, whose concentration may exceed 10^10^ cells per cm^2^ of surface, belong to the genera *Corynebacterium*, *Arthrobacter*, *Brevibacterium*, *Staphylococcus* and *Micrococcus* [1], [2], [3], [4], [5], [6], [7], [8]. However, growth of these bacteria at the surface of smear-ripened cheeses is difficult to control, which may affect the organoleptic and sanitary quality of the final product. For example, strains that are deliberately inoculated into cheese milk frequently do not establish themselves on the cheese surface [1], [3], [5], [9], [10]. The determinants that are involved in the ability of bacteria to grow at the surface of cheese are not well known. In the past, the production of smear-ripened cheeses involved a common step of “old-young smearing”, i.e., the transfer of an undefined microflora washed off from the surface of ripened cheeses to young unripened cheeses. This process favoured the selection, in a quasi-continuous manner, of microorganisms well adapted to the cheese surface. Therefore, smear-ripened cheeses are interesting examples of adaptation of microorganisms to a new habitat. We elected to sequence the genome of strain Re117, the type strain of *Arthrobacter arilaitensis*, because it is a typical species from the surface of smear-ripened cheeses [3], [5], [7], [11] and is one of the yellow-pigmented species which are believed to be responsible for colour development [12], [13]. Moreover, the availability of the genome sequences of three *Arthrobacter* strains originating from soils (*A. aurescens* TC1 [14], *A. chlorophenolicus* A6 and *Arthrobacter* sp. FB24) permits comparative genomic analyses which may help to identify genetic determinants specific to the cheese habitat and to understand the emergence of bacterial species adapted to the surface of cheese.

## Methods

### Arthrobacter strains and culture conditions

*Arthrobacter arilaitensis* strain Re117 was previously isolated from the surface of a Reblochon cheese [11]. This strain is the type strain of the species, and was deposited in the CIP public strain collection (Collection of Institut Pasteur) as strain CIP 108037. It was routinely grown at 25°C in brain heart infusion broth (BHI, Biokar Diagnostics, Beauvais, France) under aerobic conditions (rotary shaker at 150 rpm, volume of broth equivalent to 20% of the volume of the conical flask). *Arthrobacter aurescens* TC1, *Arthrobacter* sp. FB24 and *A. chlorophenolicus* A6 were kindly provided by M.J. Sadowsky, C.H. Nakatsu and J.K. Jansson, respectively. Total genomic DNA of strain Re117 was isolated according to the protocol described by Feurer et al. [3] using mechanical glass bead-based lysis followed by phenol extraction and nucleic acid precipitation by ethanol.

Carbon-source utilization tests were performed with the Biotype 100 system using the minimal growth medium 2 (bioMérieux, Marcy l′Etoile, France). The assimilation of D-Galactonic acid was tested separately by addition of 30 mM D-Galactono-γ-lactone (MP Biomedicals, Illkirch, France) to the minimal growth medium 2. Incubation of the Biotype 100 strips was performed for 6 days at 30°C under aerobic conditions. The effect of NaCl concentration on the growth of *Arthrobacter* strains was determined in brain heart infusion broth at 25°C under aerobic conditions. The maximum growth rate was determined by the slope of the plot relating ln absorbance (600 nm) versus time.

### Model cheese experiments

Strain Re117 was cultivated as described above and the yeast *Debaryomyces hansenii* 304 (local strain collection) was grown at 25°C in potato dextrose broth (Biokar Diagnostics) under aerobic conditions. Model cheeses were produced as described previously [12], except that the lactic curd was inoculated with 10^4^ cfu/g of the yeast and 10^6^ cfu/g of *A. arilaitensis* Re117. Some experimental cheeses were supplemented with 1 mg of iron per kg of curd. Iron was added using a filter-sterilized concentrated solution of FeCl_3_.6H_2_O. The inoculated cheeses were incubated for 24 h at 21°C and then at 14°C. The function of *Debaryomyces hansenii* is to raise the pH of the cheeses, thereby favouring the growth of *A. arilaitensis*. At each sampling time, three separate cheeses were sampled. Growth of the lactic acid bacteria, of *A. arilaitensis* and *Debaryomyces hansenii* was measured on MRS agar supplemented with amphotericin, on BHI agar supplemented with amphotericin, and on YGCA agar, respectively, as described previously [12].

### Genome sequencing

The complete genome sequence of *Arthrobacter arilaitensis* Re117 was determined using a mix of Sanger and new technology sequencing. Around 20× coverage of 454 GSflx reads were mixed with 4× coverage Sanger reads for the scaffolding, which was derived from a 10-kb insert fragment size library. This library was constructed after mechanical shearing of genomic DNA and cloning of the resulting fragments into plasmid pCNS (pSU18-derived). Plasmid DNAs were purified and end-sequenced (27648 reads) by dye-terminator chemistry with ABI3730 sequencers (Applied Biosystems, Foster City, USA), leading to an approximately 4-fold coverage. The sequences were assembled using Arachne 2 (www.broadinstitute.org) and validated via the Consed interface (www.phrap.org). For the finishing phases, we used primer walking of clones, PCR and *in vitro* transposition technology (Template Generation System™ II Kit; Finnzyme, Espoo, Finland), corresponding to 275, 41 and 4800 additional reads, respectively.

### Genome analysis and annotation

Genome annotation was performed with AGMIAL [13], an integrated bacterial genome annotation system. Prediction of coding sequences used the self-training gene detection software SHOW based on Hidden Markov Models (http://genome.jouy.inra.fr/ssb/SHOW/). tRNA and rRNA were detected using the tRNAscan-SE [14] and RNAmmer [15] softwares, respectively. Web-based software and databases were used to manually curate predicted genes after comparing data to public databases (COGs [16], Conserved Domains Database [17] and InterPro [18]). During the annotation process, several gene fragments were detected. The corresponding sequences were manually inspected to rule out sequencing or assembly errors. Insertion Sequences (ISs) were identified and classified using the ISfinder database (http://www-is.biotoul.fr) as described by Siguier and coworkers [19]. Transporters were classified using the TCDB (http://www.tcdb.org/) [20] and ABCISSE (http://www.pasteur.fr/recherche/unites/pmtg/abc/) [21] databases. Gram-positive signal sequences and Twin arginine transport signal sequences were predicted using SignalP (http://www.cbs.dtu.dk/services/SignalP/) [22] and TatP (http://www.cbs.dtu.dk/services/TatP/) [23].

The genome sequences of *Arthrobacter aurescens* TC1 [24] (accession number CP000474), *Arthrobacter* sp. strain FB24 (accession number CP000454), a strain isolated from a xylene and chromate enriched soil microcosm [25], and *A. chlorophenolicus* A6 (accession number CP001341), a strain capable of degrading high concentrations of 4-chlorophenol [26], were used for comparative genomic analyses. The sequence data from the strains FB24 and A6 were produced by the US Department of Energy Joint Genome Institute (http://www.jgi.doe.gov/) in collaboration with the user community. Genome comparisons were performed using Origami, an in-house tool developed for microbial genome comparison. Orthologs were defined as reciprocal best hits with an e-value lower than 10^−3^. Transposases were excluded from the analysis. Core genes were defined as orthologs shared between the four *Arthrobacter* strains. Synteny was studied using an in-house developed tool, Align, using dynamic programming to search conserved gene trains allowing gaps and “mismatches” (homology relation instead of orthology). Circular representation of the genome was produced using the Circos software [27].

### Nucleotide sequence accession number

The *A. arilaitensis* Re117 chromosome sequence has been deposited in the EMBL database under accession number FQ311875. The sequences of the *A. arilaitensis* plasmids pRE117-1 and pRE117-2 are available using accession numbers FQ311475 and FQ311476, respectively.

## Results

### General genome features and comparative genomics

The genome of *A. arilaitensis* Re117 (Table 1) consists of a circular chromosome of 3,859,257 bp (locus tag prefix: AARI), a plasmid of 50,407 bp (plasmid pRE117-1; locus tag prefix: AARI_pI) and a plasmid of 8,528 bp (plasmid pRE117-1; locus tag prefix: AARI_pII). The size of the genome is less than that of the three other available sequenced *Arthrobacter* strains (5.2, 5.1 and 5.0 Mbp for *A. aurescens* TC1, *Arthrobacter* sp. FB24 and *A. chlorophenolicus* A6, respectively), which were all isolated from soil environments. 2155 of the *A. arilaitensis* protein-encoding genes (62.7%) were assigned a putative function. The G+C content is 59.2%, less than that of the other *Arthrobacter* genomes (62.4, 65.4 and 66.0% for *A. aurescens* TC1, *Arthrobacter* sp. FB24 and *A. chlorophenolicus* A6, respectively).

**Table 1:** General features of the *A. arilaitensis* Re117 genome.

Feature	Value
Chromosome G + C content (mol%)	59.2
Chromosome size (bp)	3,859,257
Plasmid pRE117-1 size (bp)	50,407
Plasmid pRE117-2 size (bp)	8,528
Total genome size (bp)	3,918,192
	
Total no. of genes	3518
No. of rRNA genes	18
No. of tRNA genes	64
No. of protein-encoding genes	3436
No. (%) of genes with function prediction	2155
No. (%) of genes without function prediction	1281
No. (%) with similarity	874
No. (%) without similarity	407
Average gene length (bp)	950
Coding density (%)	83.1
No. of pseudogenes	123

Plasmid pRE117-1 is predicted to encode 47 proteins, most (28) with no assigned function. Approximately 10% of the plasmid is constituted of insertion sequences. pRE117-1 also encodes three DNA mobilization proteins (AARI_pI00200-00220) and a putative secreted peptidase (AARI_pI00350). Genes AARI_pI00010-00050 encode a single-stranded DNA-binding protein and proteins of unknown function. They are highly conserved (73 to 95% amino acid identity) and show the same order as in plasmid pAG1 of *Corynebacterium glutamicum* 22243 [28]. Ten pRE117-1 proteins had a best BLAST hit with other *Arthrobacter* plasmid proteins, among which were five from plasmid FB24-2 of *Arthrobacter* sp. FB24 [25]. Plasmid pRE117-2 is predicted to encode 13 proteins, of which nine are hypothetical. Two (AARI_pII00060 and AARI_pI00070) are predicted to be mobilization proteins.

**Figure 1:**
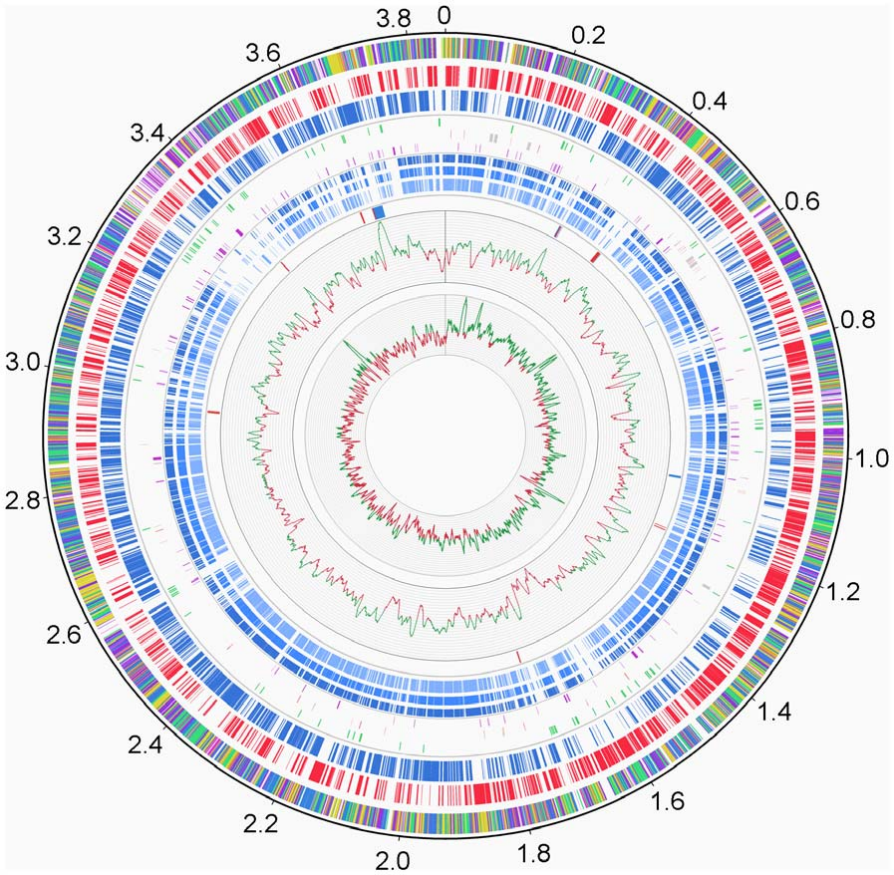
*Arthrobacter arilaitensis* Re117 genome atlas. The outermost circle (circle 1) represents the scale in Mbp. Circle 2 represents the functional category of the CDSs: green, cell envelope and related processes; blue, intermediary metabolism; yellow, information pathways; orange, other functions; dark purple, proteins of unknown function that are similar to other proteins; light purple, proteins of unknown function without similarity to other proteins. Circles 3 and 4 represent CDSs (excluding transposases) on positive (red) and negative (blue) strands. Circle 5 represents the insertion sequences (in green). Circle 6 represents rRNA (grey) and tRNA (red) genes. Circle 7 represents the pseudogenes (excluding transposase pseudogenes). Circles 8, 9 and 10 represent CDSs (in blue) orthologs (predicted as defined in section “Genome analysis and annotation”) with genes from three environmental *Arthrobacter* strains: *A. chlorophenolicus* A6 (circle 8), *Arthrobacter* sp. FB24 (circle 9) and *A. aurescens* TC1 (circle 10). Circle 11 represents genes involved in siderophore biosynthesis and export (blue) and in Fe^3+^/siderophore complexes import (red). Circle 12 and 13 represent the G+C content and GC skew (G−C)/(G+C), respectively, each plotted using a 10-kb window.

Six rRNA operons were identified. These are organized in the order 16S-23S-5S and located on the same replichore. Three are near the replication origin (Figure 1). The sequences of the six 5S rRNA from *A. arilaitensis* Re117 were 100% identical, whereas one mismatch was observed for one of the 16S rRNA (AARI_36520) and for one of the 23S rRNA (AARI_36350; a sequence variation of <0.1%). Sixty four tRNA genes were detected in *A. arilaitensis* Re117 (Figure 1) by the tRNA-scan progam (Table S1). Of the 123 putative pseudogenes (∼3.5% of the total number of genes) detected, 21 were associated with insertions of ISs, 40 originated from genes of unknown function, 21 from putative transporters and 9 from putative transcriptional regulators (Table S2). *Arthrobacter arilaitensis* Re117 is equipped with a full complement of genes to carry out repair of DNA lesions (Table S3) and with three complete type I restriction modification (R/M) systems (AARI_22810-22830, AARI_33320-33340, and AARI_pI00090-00110). A Sec-dependent protein secretion pathway (AARI_17430-17450, AARI_19000, AARI_20960, AARI_23280, AARI_23680 and AARI_34330) and a twin-arginine translocation (Tat) pathway (AARI_16440, AARI_16450 and AARI_2163) are present. The *A. arilaitensis* genome encodes 356 predicted transport proteins (Table S4), which represent 10.4% of the CDSs. This percentage is less than in *A. aurescens* TC1, where 12.1% of the CDSs were assigned to transport proteins [24].

Of the 2727 *A. arilaitensis* Re117 CDSs that produced a best BLAST hit above-threshold (e-value lower than 10^−3^ and overlap of more than 80%), 2538 (93%) had their best BLAST hit with CDSs from bacteria belonging to the phylogenetic order *Actinomycetales.* Nearly half (1210) of the hits are seen with one of the three other available sequenced *Arthrobacter* strains (Figure S1). A global comparative analysis was performed to assess both the common and unique CDSs of each of the four sequenced *Arthrobacter* strains (Figure S2). A total of 1545 CDSs are conserved in the four *Arthrobacter* genomes and 1256 CDSs are specific for *A. arilaitensis* Re117. A large number (746) of CDSs were absent in *A. arilaitensis* Re117 but present in all three of the environmental *Arthrobacter* genomes. The absence of these genes in *A. arilaitensis* Re117 may reflect gene loss in adaptation to growth on cheese surface. The gene distributions were compared by classifying the predicted protein products into major functional categories, according to the COG protein classification scheme (Figure S3). It is noteworthy that the percentage of *A. arilaitensis* genes in the COG group G (carbohydrate transport and metabolism) is much lower than in the environmental *Arthrobacter* strains. Approximately 20% of the genes common to the environmental strains but absent in *A. arilaitensis* belong to this COG group.

Synteny plots show the presence of large blocks of similarity between the *A. arilaitensis* chromosome and the chromosomes of *A. aurescens* TC1, *A. Arthrobacter* sp. FB24, or *A. chlorophenolicus* A6 (Figure S4). Three blocks corresponding to large chromosome rearrangements are present near the putative replication terminus. For the CDSs corresponding to the rightmost region of the plots (about 25% of the *A. arilaitensis* CDSs), the overall degree of synteny was very low. The circular representations (Figure S5) of the chromosomes of the three environmental *Arthrobacter* strains in which the orthologs present in *A. arilaitensis* Re117 are indicated show that segments of varying sizes are absent in *A. arilaitensis*. These represent up to 70 kbp.

### Insertion sequences

One hundred and nine complete and 35 partial IS copies representing 12 families comprising 4.92% of the *A. arilaitensis* chromosome were identified (Figure 1, Table S5 and Table S6). This was considerably more than observed in the chromosomes of any of the *Arthrobacter* species isolated from the soil (*A. aurescens* TC1: 10 complete and 3 partial, 0.33%; *A. chlorophenolicus* A6: 1 complete and 2 partial, 0.08%; *Arthrobacter* sp. FB24: 6 complete and 8 partial, 0.4%) (Table S5). The *A. arilaitensis* ISs are not evenly distributed along the chromosome but tend to be clustered in several regions. The two most striking are located between positions 3.25 and 3.44 Mbp (24 of the 109 complete copies and 14 of the 35 partial copies) and the second between positions 1.47 and 1.72 Mbp (24 of the 109 complete copies and 3 partial copies). The first includes a regional change in GC skew suggesting either a recent insertion or inversion whereas the second includes a region with GC content significantly different from that of the surrounding sequences suggesting a recent horizontal aquisition. Unsurprisingly several ISs were also identified in certain of the larger *Arthrobacter* plasmids.

Certain clusters of ISs were found inserted one within the other like “russian dolls” as has been observed in several other bacterial genomes. Two such intercalated IS couples (IS*Aar16* inserted into IS*Aar15* and IS*Aar18* inserted into IS*Aar17*) appear to have retained transposition activity since they are present as identical sequences in two copies at different locations in the genome.

In addition, members of the Tn*3* and Tn*554* transposon families were identified in the soil bacteria but are not present in *A. arilaitensis*. *Arthrobacter aurescens* TC1 carries a complete copy of an Tn*3* derivative in the chromosome which includes two genes (a putative glyoxalase and a hypothetical protein), and a complete and partial copy of IS*1071* (also a Tn*3* derivative) in pTC1. Interestingly, IS*1071* is also found in other soil bacteria such as *Alcaligenes* sp. BRC60, *Burkholderia cepacia* st 2A, *Comamonas testosteroni*, *Ralstonia eutropha* and *Ralstonia metallidurans* CH34. An additional Tn*3* family member of 26712 bp carrying an operon of 23 ORFs, which include heavy metal resistance genes, is found as identical copies in all three *Arthrobacter* sp. FB24 plasmids in different sequence environments.

### Gene regulation

The genome of *A. arilaitensis* Re117 encodes 253 proteins with possible function in gene regulation (one-component regulators, two-component regulators, sigma and anti-sigma factors), representing 7.4% of the CDSs. One hundred and ninety nine of the regulatory proteins correspond to one-component regulators (Table S7). Nine LacI-family regulators are present in strain Re117, whereas *A. aurescens* TC1, *Arthrobacter* sp. FB24 and *A. chlorophenolicus* A6 contain respectively 30, 28 and 21 regulators belonging to this family (based on the number of proteins matching the pfam PF00356 Hidden Markov Model). LacI-family regulators are frequently involved in the control of degradative pathways, and many of them recognize sugar-inducers [29]. The lower occurrence of these regulators in strain Re117 suggests a lower degradative capacity compared to the environmental *Arthrobacter* strains. Two WhiB family protein (AARI_07720 and AARI_12070) are present in *A. arilaitensis* Re117. This family represents a group of transcriptional activators specific for actinobacteria and absent from all other sequenced bacterial genomes [30]. WhiB protein family members were postulated to function in cell division and septation of mycobacteria [31] and in differentiation of streptomycetes [32]. An iron-dependent repressor IdeR, which mediates global iron regulation in high-GC-content Gram-positive bacteria [33], is present in *A. arilaitensis* Re117 (AARI_05610).

The number of two-component system (TCS) signal transduction genes (Table S7) is close to that of *A. aurescens* TC1 (46 *vs*. 55). The stimuli sensed by these TCSs are unknown. The genes encoding the TCS MtrA/MtrB are followed by *lpqB*. This cluster is conserved in all actinobacteria sequenced to date with only a single exception, the human intracellular pathogen *Tropheryma whipplei* [34]. MtrAB may have a role in the regulation of osmoprotection, cell envelope homeostasis and cell cycle progression, and the lipoprotein LpqB may be an auxiliary protein involved in the MtrAB signal transduction pathway. Bacterial sigma factors belong to two protein families: the σ54 and the σ70 families. The σ70 family can be further divided into four phylogenetic groups, with the extracytoplasmic function (ECF) group being the largest and most diverse [35]. ECF sigma factors offer a convenient mechanism for transcriptional regulation in response to specific environmental signals. In general, bacteria with complex lifestyles involving different habitats have more ECF sigma factors than bacteria with simple lifestyles living in stable niches [36], [37]. The number of σ70-family sigma factors, estimated as the number of proteins matching the pfam PF04542 Hidden Markov Model, was much lower in *A. arilaitensis* Re117 (six CDSs) than in the environmental *Arthrobacter* strains (19 CDSs in *A. aurescens* TC1, 11 in *Arthrobacter* sp. FB24, and 12 in *A. chlorophenolicus* A6), probably reflecting the more stable habitat of *A. arilaitensis*, due to its adaptation to cheeses. A significant number (34%) of *A. arilaitensis* proteins with possible function in gene regulation have no ortholog in any of the three environmental *Arthrobacter* strains.

### Metabolism of amino acids

The biosynthetic pathways for all amino acids appear complete, with the exception of the asparagine biosynthesis pathway, for which no candidate for ammonia-dependent asparagine synthetase (EC 6.3.1.1) or asparagine synthase (EC 6.3.5.4) was identified. Genes encoding tRNA synthetases for glutamine and asparagine were not found. It is likely that the GatABC system (AARI_08740-08760) is used to produce glutamine and asparagine from tRNAs charged with glutamate and aspartate.

A bifunctional proline dehydrogenase/pyrroline-5-carboxylate dehydrogenase, which catalyses oxidation of proline to glutamate using a membrane-bound quinone and NAD as electron acceptor [38], is present (AARI_26900). On Biotype 100 strips, *A. arilaitensis* Re117 is able to use proline as a carbon source (Table S8). Caseins are very rich in proline: e.g. 17 of the 199 residues in αs1-casein and 35 of the 209 residues in β-casein are proline, and proline is one of the main free amino acids in cheese [39], [40]. This compound may thus constitute a substrate for *A. arilaitensis* growth in cheese.

*Arthrobacter arilaitensis* Re117 encodes proteins with possible function in tyrosine degradation. We identified enzymes probably involved in catabolism of homoprotocatechuic acid to succinate and pyruvate (AARI_06160, AARI_06170, AARI_07830, AARI_13280, AARI_13290, AARI_13310, AARI_26540 and AARI_31350). The genes encoding two of the enzymes of the degradative pathway (e.g. 4-hydroxyphenylpyruvate oxidase and 4-hydroxyphenylacetate 3-monooxygenase) were not located, but such activities are probably present, because *A. arilaitensis* Re117 is able to use tyrosine as a carbon source. This pathway is present in several Gram-positive bacteria [41], including an *Arthrobacter* sp. strain [42]. As cheese frequently contains high amounts of tyrosine [43], this compound may constitute a substrate for the growth of *A. arilaitensis* in cheese.

*Arthrobacter arilaitensis* Re117 contains an NAD-specific glutamate dehydrogenase (EC 1.4.1.2; AARI_20900) which may be used to produce energy by conversion of glutamate to α-ketoglutarate, an intermediate of the TCA cycle, concomitantly generating NADH. *Arthrobacter arilaitensis* Re117 is able to use glutamate as a carbon source on Biotype 100 strips. This amino acid is another major amino acid from casein (27 to 38 glutamate and glutamine residues per molecule, depending on the casein type) and among the major free amino acids in cheese [40].

Volatile sulfur compounds are major flavour compounds in many types of cheeses [44]. They arise primarily from degradation of L-methionine to methane thiol by the cheese ripening microorganisms. This thiol is a common precursor of a variety of odorant sulfur-containing compounds [45]. The ability of some cheese ripening bacteria to produce high amounts of volatile sulfur compounds has been attributed to the presence of L-methionine-γ-lyase (EC 4.4.1.11), cystathionine gamma-lyase (EC 4.4.1.1) or cystathionine beta-lyase (EC 4.4.1.8) activities [46], [47], [48]. However, no candidate genes for these enzymes was identified in *A. arilaitensis Re117*.

4-Aminobutyrate (GABA) is a four-carbon non-protein amino acid known to occur in cheese [49]. The *A. arilaitensis* Re117 genome encodes a 4-aminobutyrate transaminase (EC 2.6.1.19; AARI_24090) and three succinate-semialdehyde dehydrogenases (EC 1.2.1.16; AARI_07830, AARI_26540 and AARI_31350). These enzymes convert 4-aminobutyrate into succinate, an intermediate of the TCA cycle. Although *A. arilaitensis* Re117 cannot use 4-aminobutyrate as carbon source on Biotype 100 strips, we cannot exclude that it is able to catabolize this compound during its growth in cheese. The degradative pathways for lysine, arginine, cysteine, tryptophan, leucine, phenylalanine and valine are absent or incomplete (Table S8).

The pathways for histidine, glycine, threonine, serine, alanine, isoleucine and aspartate are present, but *Arthrobacter arilaitensis* Re117 is not able to assimilate histidine on Biotype 100 strips.

### Catabolism of biogenic amines

Cheese may be a significant source of biogenic amines such as tyramine, histamine, cadaverine, tryptamine, phenylethylamine and putrescine [50]. If present at a high concentration, these compounds may be toxic and generate an undesired flavour [40]. They result from the catabolism of amino acids during ripening by the microorganisms that are present. A putative primary-amine oxidase (EC 1.4.3.21) is present in *A. arilaitensis* Re117 (AARI_26060). It shows a high level of identity (62% at the amino acid level) with the phenylethylamine oxidase from *A. globiformis* IFO 12137. This enzyme catalyses oxidative deamination of phenylethylamine and tyramine [51]. A putative putrescine oxidase (EC 1.4.3.10) was also identified (AARI_27810) which shows a high level of identity (62% at the amino acid level) with the putrescine oxidase from *Micrococcus rubens* IFO 3768. This enzyme catalyses the oxidative deamination of putrescine, and, at a lower rate, of cadaverine and spermidine [52]. A putative polyamine ABC transport system has also been identified (AARI_25840-25880). The presence of enzymes and importers involved in the catabolism of biogenic amines indicates that some strains of *A. arilaitensis* may contribute to the reduction of the content of biogenic amines in cheeses as observed for *Brevibacterium linens* [53].

### Central carbohydrate metabolism

All TCA cycle genes are present. Unlike most bacteria, conversion of malate to oxaloacetate is catalysed by a malate dehydrogenase functioning with a quinone acceptor (EC 1.1.99.16, AARI_13630) rather than with NAD (E.C. 1.1.1.37). Using a quinone as acceptor instead of NAD may allow an organism to attain a high tricarboxylic acid cycle flux independently of the NADH/NAD and malate/oxaloacetate ratios [54]. Use of a quinone acceptor may also be of importance for a robust citric acid cycle flux under adverse conditions such as low electron acceptor concentrations and limiting concentrations of carbon sources. Genes encoding the malate synthase (EC 2.3.3.9) and isocitrate lyase (EC 4.1.3.1), which constitute the glyoxylate bypass, are present, with two paralogs for the malate synthase (AARI_03300 and AARI_33430). The glyoxylate bypass is an alternate route which bypasses the CO_2_-evolving steps of the tricarboxylic acid cycle thus permitting the utilization of fatty acids or acetate in the form of acetyl-CoA, as the sole carbon source. It is an essential anaplerotic pathway (replenishment of the TCA cycle intermediates) in several microorganisms, such as *Corynebacterium glutamicum* growing on acetate [55]. Genes encoding enzymes from other anaplerotic reactions, i.e. phosphoenolpyruvate carboxykinase (EC 4.1.1.32) and pyruvate carboxylase (EC 6.4.1.1) are also present (AARI_05030 and AARI_11990) as are genes representing the complete glycolysis, gluconeogenesis and pentose phosphate pathways.

*Arthrobacter arilaitensis* is an aerobic respiratory bacterium and thus produces most of its ATP by oxidative phosphorylation. Several dehydrogenases from the respiratory chain are present. These include a type II NADH oxidase (EC 1.6.99.3; AARI_07440), a succinate dehydrogenase complex (EC 1.3.99.1; AARI_07200-07230), and D-lactate (EC 1.1.1.28; AARI_22340), L-lactate (EC 1.1.1.28; AARI_31060), pyruvate (EC 1.2.2.2; AARI_17870), and glycerol-3-phosphate (EC 1.1.99.5; AARI_17030) dehydrogenases. As indicated above, a malate dehydrogenase (EC 1.1.99.16, AARI_13630) and a proline dehydrogenase (EC 1.5.99.8; AARI_26900) functioning with quinone acceptors are also present. Orthologs of all these genes, except for the D-lactate dehydrogenase, are found in the genomes of the environmental strains *A. aurescens* TC1, *A. chlorophenolicus* A6 and *Arthrobacter* sp. FB24.

D- and L-lactate are present in most cheeses at the beginning of ripening and the presence of quinone dependent D- and L-lactate dehydrogenases may allow *A. arilaitensis* to produce energy from these substrates. *Arthrobacter arilaitensis* Re117 is able to use lactate as a carbon source on Biotype 100 strips (Table S9). Interestingly, two putative lactate transporters are present in *A. arilaitensis* (AARI_27940 and AARI_28020), but have not been identified in *A. aurescens* TC1 and *A. chlorophenolicus* A6 and only a single example is present in *Arthrobacter* sp. FB24 (pfam family PF02652).

As in *Corynebacterium glutamicum* [56], [57], *A. arilaitensis* Re117 contains putative genes for three enzymes which couple electron transfer to the generation of an electrochemical proton gradient across the cytoplasmic membrane, i.e. the cytochrome bc1 complex (AARI_16650-16670), the cytochrome aa3 oxidase (AARI_16640 and AARI_16690-16710) and the cytochrome bd oxidase (AARI_20160-20170). The genes encoding the eight subunits of the H^+^-transporting two-sector ATPase (EC 3.6.3.14), an enzyme essential for ATP generation by oxidative phosphorylation, are organized in the *atpBEFHAGDC* operon (AARI_12680-12750).

**Figure 2:**
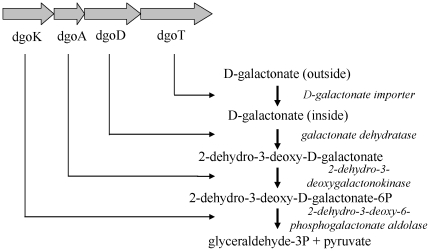
Genes probably involved in the catabolism of D-galactonate by *A. arilaitensis* Re117.

In comparison to the three environmental *Arthrobacter* strains, *A. arilaitensis* has a much lower capacity for growth on different carbohydrates (Table S9). Gene candidates were identified for the enzymes required for the catabolism of D-gluconate, D-cellobiose, maltose, ribose, sucrose, D-galactose (Leloir pathway) and lactose. D-galactose and lactose may be present in some cheeses at the beginning of ripening. It is unable to grow on: fructose (probably due to a truncation of the fructose-specific phosphotransferase system IIABC components; pseudogene AARI_02340), N-acetyl-glucosamine (probably due to a frameshift mutation of the glucosamine-6-phosphate isomerase; pseudogenes AARI_08670 and AARI_08680) and D-mannose and mannitol (resulting from the absence of the identifiable catabolic enzymes). Interestingly, a cluster of four genes involved in D-galactonate catabolism is present in *A. arilaitensis* (Figure 2). This is absent in the three environmental *Arthrobacter* strains and probably results from a horizontal gene transfer as the closest orthologs are found in Gram-negative species (from 44 to 81% amino acid identity with the orthologs from *Pseudomonas fluorescens*). DgoT (AARI_31720) is a D-galactonate importer, and DgoA (AARI_31740), DgoD (AARI_31730) and DgoK (AARI_31750) are the three enzymes that convert D-galactonate into pyruvate and D-glyceraldehyde 3-phosphate. *Arthrobacter arilaitensis* Re117 grows on D-galactono-γ-lactone (hydration of this compound occurs spontaneously, generating D-galactonate), confirming that the D-galactonate catabolic pathway is functional in this strain (Table S9). It is noteworthy that there are many fewer ABC-type sugar import systems in *A. arilaitensis*, which may account for its low capacity for growth on various carbohydrates. Indeed, the total number of COG hits corresponding to ABC-type sugar import components is 25, whereas 104, 102 and 79 hits are found for *A. aurescens* TC1, *Arthrobacter* sp. FB24 and *A. chlorophenolicus* A6, respectively (Table S10). The more limited carbohydrate catabolic activity of *A. arilaitensis* compared to the environmental *Arthrobacter* strains may be due to its adaptation to cheese, which contains only a limited number of carbohydrates. The inability of *A. arilaitensis* Re117 to grow with citrate as a carbon source is probably due to mutation of the CitAB two-component system (Table S2). Indeed, in *Corynebacterium glutamicum*, CitAB is required for citrate utilization [58]. Citrate is a constituent of milk, but it is absent in most cases when the ripening bacteria begin to grow at the surface of cheese, due to the presence of mesophilic citrate-utilizing lactic acid bacteria that grow at the beginning of cheese making.

### Proteolysis

In all ripened cheese varieties, proteolytic enzymes contribute to flavour and taste development and are responsible for changes in body and texture characteristics. In general, protein degradation may occur by extracellular enzymes secreted from the microorganisms throughout growth or by intracellular enzymes released upon lysis of microorganisms. While excreted proteinases might be expected to be involved in casein degradation, enzymes released upon lysis are more likely to be peptidases and are expected not to be directly involved in casein degradation but rather in degradation of peptides generated by the hydrolytic action of proteinases. Smacchi et al. [59] characterized an extracellular protease from a cheese-borne *A. nicotianae*, a species related to *A. arilaitensis* [11]. This enzyme is active at the pH, temperature and NaCl concentration typical of the surface of smear-ripened cheeses and may therefore contribute to the proteolysis of these cheeses during ripening.

The genome of *A. arilaitensis* Re117 encodes at least 51 proteins with putative protein degradation functions (Table S11). For 36 of the corresponding genes, orthologs were found in *A. aurescens* TC1, *Arthrobacter* sp. FB24 and *A. chlorophenolicus* A6 (nine were specific for *A. arilaitensis* Re117). A signal peptide (signal peptidase I cleavage site) was predicted in eight of these proteins.

### Carotenoid biosynthesis

A cluster of carotenoid biosynthesis genes is present in *A. arilaitensis* Re117 (AARI_13710-13770) but absent in *Arthrobacter* sp. FB24 and *A. chlorophenolicus* A6. It is present in *A. aurescens* TC1 and shows a similar organization, but the homolog of AARI_13740 has been annotated as a pseudogene (AAur_0317). Carotenoids are yellow-, orange-, or red-coloured pigments formed by the condensation of isoprenyl units. One of their main functions is to protect the cells from harmful oxygen radicals [60]. It has been shown that yellow-pigmented bacteria such as *A. arilaitensis* and *Microbacterium gubbeenense* produce predominant pigments in smear-ripened cheeses [61].

Interestingly, strains Re117 and TC1 form yellow colonies on agar plates in contrast to FB24 and A6, which form cream colonies. Idi (AARI_13770), CrtE (AARI_13710), CrtB (AARI_13720) and CrtI (AARI_13730) catalyse the biosynthesis of lycopene from isopentenyl diphosphate. CrtYe (AARI_13740), CrtYf (AARI_13750) and CrtEb (AARI_13760) are proteins whose counterparts in *Corynebacterium glutamicum* (from 44 to 54% identity) catalyze the biosynthesis of the C_50_ carotenoid decaprenoxanthin from lycopene. Therefore, the yellow pigment produced by *A. arilaitensis* Re117 may belong to the C_50_-subfamily, whose production has been described only in a few bacteria [62].

### Oxidative and osmotic stress tolerance, carbon storage

*Arthrobacter arilaitensis* Re117 contains genes encoding proteins involved in oxidative stress tolerance, including three catalases (AARI_02500, AARI_07180 and AARI_14710), a superoxyde dismutase (AARI_19040), two thiol-specific antioxidant proteins (AARI_09110 and AARI_15340) and two peroxiredoxins (AARI_13660 and AARI_31340). Glycine betaine is a very efficient osmolyte, which is accumulated in response to osmotic stresses. The genome contains a homolog of *codA* (AARI_07270), which encodes a choline oxidase (EC 1.1.3.17) that catalyses the two steps of glycine betaine synthesis from choline in *A. globiformis* [63]. This gene is clustered with *betB* and *betT*, which encode a betaine-aldehyde dehydrogenase (EC 1.2.1.8; AARI_07260) and a high-affinity choline transporter (AARI_07250), respectively. The genes encoding two of the three enzymes that catalyse the degradation of glycine betaine to glycine (EC 2.1.1.5 and EC 1.5.99.2) were not identified. For the third enzyme, the sarcosine oxidase (EC 1.5.3.1), which is composed of four subunits, a very high level of identity (from 98 to 100% at the amino acid level) was observed with the homologs present in the Gram-negative emerging opportunist pathogen *Stenotrophomonas maltophilia*.

Fifteen genes involved in the transport of glycine betaine and related osmolytes were identified in Re117 by the manual annotation. Eleven correspond to ABC transport components (TC 3.A.1.12) and the others to transporters of the betaine/carnitine/choline transporter (BCCT) family (TC 2.A.15). This number is lower in the genomes of environmental strains *A. aurescens* TC1, *Arthrobacter* sp. FB24 and *A. chlorophenolicus* A6 (from six to nine genes, Table S12) and seven of the *A. arilaitensis* genes have no ortholog in any of the three soil-originating strains. Interestingly, growth of *A. arilaitensis* is less affected by salt concentration than that of the environmental *Arthrobacter* strains (Figure S6). The presence of a high salt concentration at the surface of smear-ripened cheeses may have favoured selection of efficient osmoprotectant transporter systems.

Trehalose is a non-reducing sugar that seems to play a major physiological role in energy storage in actinobacteria and as an environmental protectant against various stresses such as desiccation or dehydration, external osmolarity fluctuations, heat, cold and oxidation [64]. *Arthrobacter arilaitensis* Re117 can use trehalose as carbon source on Biotype 100 strips. Trehalose biosynthesis by this organism probably occurs via trehalose-6-phosphate synthase (EC 2.4.1.15; AARI_05410) and trehalose-6-phosphate phosphatase (EC 3.1.3.12; AARI_05400). The environmental strains *Arthrobacter* sp. FB24, *A. chlorophenolicus* A6 and *A. aurescens* TC1 also have this pathway but, in addition, they contain the genes involved in biosynthesis of trehalose from maltodextrin (maltooligosyl-trehalose synthase EC 5.4.99.15 and maltooligosyl trehalohydrolase EC 3.2.1.141) and from maltose (trehalose synthase EC 5.4.99.16).

Glycogen is a major intracellular carbon source reserve polymer. It is accumulated under conditions of limiting growth when an excess of carbon source is available and other nutrients are deficient [65]. In *A. arilaitensis* Re117, the genes encoding the three proteins GlgA (AARI_18970), GlgB (AARI_18950) and GlgC (AARI_18960) which catalyze glycogen biosynthesis, form a putative operon with the gene encoding GlgP (AARI_18970) which participates in glycogen degradation. In the three environmental *Arthrobacter* strains, *glgP* and *glgB* form a distinct cluster from *glgA* and *glgC*.

### Catabolism of lipids

Cow's milk contains on average 35 g/l of lipids, mainly triglycerides. These lipids are a source of fatty acids. Fatty acids have a direct effect on cheese flavour but it is mainly their breakdown that leads to accumulation of compounds having low olfactive thresholds (e.g., alcools, ketones, lactones, esters and thioesters) significant in cheese flavour perception [49]. In smear-ripened cheeses, free fatty acids may be present at a concentration higher than 4 g/kg [66] and it is likely that the surface microflora makes a significant contribution to lipolysis [67], [68], [69]. Fatty acids constitute an important energy source for growth of aerobic ripening microrganisms. *Arthrobacter arilaitensis* Re117 encodes 17 proteins with putative lipase or esterase activity (Table S13) including a secretory triacylglycerol lipase (AARI_04500), which may have a higher contribution to the ripening of smear-ripened cheeses than intracellular lipases or esterases. Furthermore, *A. arilaitensis* Re117, which can use glycerol as carbon source on Biotype 100 strips, has a cluster of genes for the aerobic catabolism of glycerol (AARI_17010-17030). These encode a glycerol uptake protein, a glycerol kinase (EC 2.7.1.30) and a glycerol-3-phosphate dehydrogenase (EC 1.1.99.5). The genes encoding the characteristic enzymes of the β-oxidation pathway are present. Interestingly, five of the eleven predicted acyl-CoA dehydrogenases and four of the eleven predicted fatty acid—CoA ligases have no ortholog in any of the environmental strains *A. aurescens* TC1, *A. chlorophenolicus* A6 and *Arthrobacter* sp. FB24 (Table S14). This may be the result of adaptation of *A. arilaitensis* to the fatty acids present in cheeses.

The catabolism of odd-chain-length fatty acids yields propionyl-CoA in addition to acetyl-CoA. This compound may be catabolized by the methylcitrate cycle. The genes encoding the characteristic enzymes of this cycle were identified by manual annotation (cluster AARI_11690-11710), i.e. the methylcitrate synthase (EC 2.3.3.5; AARI_11690), the methylcitrate dehydratase (EC 4.2.1.79; AARI_11710) and the methylcitrate lyase (EC 4.1.3.30; AARI_11700).

### Iron metabolism

Iron is an essential mineral for nearly all organisms. It is used in a variety of cofactors, e.g. heme moieties and iron-sulfur clusters, making it essential in metabolic processes such as respiration and TCA cycle. However, the amount of bioavailable iron is very low, especially at neutral pH and in the presence of oxygen, due to formation of insoluble iron oxyhydroxide compounds, resulting in extremely low concentrations of free Fe^3+^. The main mechanism used by bacteria for iron acquisition is the synthesis of siderophores, which are strong Fe^3+^ chelators. The Fe^3+^/siderophore complexes are transported into the cell by ABC transporters. During manual annotation of the *A. arilaitensis* Re117 genome, 30 genes were assigned to components of Fe^3+^/siderophore transporters (Table 2). This is higher than for the soil-originating strains *A. aurescens* TC1 (18 genes), *Arthrobacter* sp. FB24 (4 genes) and *A. chlorophenolicus* A6 (11 genes). Furthermore, 20 of the *A. arilaitensis* genes have no ortholog in any of the three environmental strains. The *A. arilaitensis* Fe^3+^/siderophore transport components apparently form nine different transport systems. Eighteen components belong to the ISVH (iron-siderophores, vitamin B12 and hemine) family [70], which constitutes the second largest ABC family in *A. arilaitensis* Re117 (Table S15). The other Fe^3+^/siderophore transport components (AARI_03920-03930) belong to the DPL family, SID (siderophore uptake) subfamily. This subfamily is constituted of importers composed of fused permease and ATPase components which have been characterized in *Yersinia pestis* [71]. The closest orthologs of AARI_03920 and 03930 are found in a Gram-negative strain, *Marinomonas* MWYL1 (50 and 52% identity at the amino acid level).

**Table 2:** Genes assigned to Fe^3+^/siderophore transporters in the *A. arilaitensis* genome.^a^

		*A. arilaitensi*s Re117		*A. aurescens* TC1		*Arthrobacter* sp. FB24		*A. chlorophenolicus* A6	
Function	COG no	number	Locus tag AARI_	number	Locus tagAaur_	number	Locus tagArth_	number	Locus tagAchl_
ABC-type Fe^3+^/siderophore transport system, ATPase component	1120, 4604	5	03940, 10930, 26330, 30190, 32480,	5	0293, 0389, 0557, 3763, 3854	1	3932	3	3005, 3732, 3784
ABC-type Fe^3+^/siderophore transport system, permease component	609, 4605, 4609, 4779	14	02850, 02860, 03950, 03960, 10920, 15030, 15040, 26340, 26350, 30200, 32460, 32470, 32760, 32770	9	0375, 0387, 0388, 0558, 2017, 3764, 3765, 3855, 3856	2	3933, 3934	5	3007, 3733, 3734, 3785, 3786
ABC-type Fe^3+^/siderophore transport system, substrate binding component	614, 4592,4607	9	02870, 03970, 03980, 10870, 15020, 26370, 30210, 32450, 32790	4	0560, 2016, 3766, 3857	1	0373	3	3006, 3735, 3787
ABC-type Fe^3+^/siderophore transport system, fused permease and ATPase component	1132	2	03920, 03930	0	/	0	/	0	/
**Total numbers:**		**30**		**18**		**4**		**11**	

a The *A. arilaitensis* genes having no ortholog in any of the three environmental *Arthrobacter* strains are underlined.

The AARI_09550-09570 cluster is probably involved in biosynthesis of a hydroxamate siderophore. The protein encoded by AARI_09560 shares homology (33% identity) with IucD, a L-lysine 6-monooxygenase (E.C. 1.14.13.59) from *Escherichia coli*, which catalyses the first step of the biosynthesis of the hydroxamate siderophore aerobactin. AARI_09550 encodes a putative siderophore biosynthesis protein of the IucA/IucC family (Pfam family PF04183) and AARI_09570 shares homology with *rhbB* (36% identity at the amino acid level), which is thought to encode a decarboxylase involved in the synthesis of a *Sinorhizobium meliloti* hydroxamate siderophore [72]. Homologs are also found in a single cluster in *A. aurescens* TC1, *Arthrobacter* sp. FB24 and *A. chlorophenolicus* A6. In addition, a putative IdeR (iron-dependent regulator) binding site (present in promoters of IdeR-regulated genes) [73] is present upstream of the AARI_09550-09570 cluster (sequence TTTGGTCAGGCTCACCTAA, position -32 relative to the translation start site of AARI_09570).

**Figure 3:**
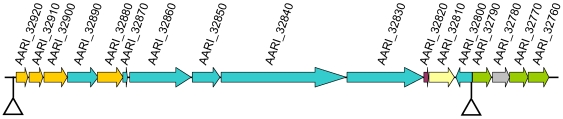
Catechol-type siderophore gene cluster in *A. arilaitensis* Re117. Genes are coloured as categorized: non-ribosomal siderophore peptide synthetase components and accessory proteins in blue, other siderophore biosynthesis proteins in orange, MbtH-like protein in dark purple, siderophore exporter in yellow, Fe^3+^/siderophore transport components in green and siderophore-interacting protein in grey. Triangles indicate the presence of putative IdeR (iron-dependent regulator) binding sites.

A catecholate or mixed catecholate-hydroxamate siderophore is presumably produced by *A. arilaitensis*. In contrast to *A. aurescens* TC1, *Arthrobacter* sp. FB24 and *A. chlorophenolicus* A6, the *A. arilaitensis* genome contains genes involved in the conversion of chorismate to the catecholate siderophore precursor 2,3-dihydroxybenzoate (AARI_32900-32920). The same gene cluster contains six non-ribosomal siderophore peptide synthetase components (AARI_32830-32870 and AARI_32890), which may be required for the modification of the basic siderophore structure (Figure 3). It also contains a gene encoding a putative L-ornithine 5-monooxygenase (AARI_32880), involved in the biosynthesis of pyoverdine, a mixed catecholate-hydroxamate siderophore produced by *Pseudomonas aeruginosa* [74], a MbtH-like protein (AARI_32820; Pfam family PF03621) which is a family of proteins found in many siderophore biosynthesis clusters, and a siderophore exporter (AARI_32810). The Fe^3+^/siderophore transport components AARI_32760-32770 and AARI_32790 are located in the same gene cluster and presumably transport the siderophore whose biosynthesis depends on the genes of this cluster. AARI_32780 is a siderophore-interacting protein that may be involved in removal of iron from the Fe^3+^/siderophore complex. A putative IdeR binding site is present upstream of AARI_32920 (sequence TTACATGAGGCTAACCTAA, position -58 relative to the translation start site) and another upstream of AARI_32790 (sequence TTACCATAGGCTACCCTTA, position -71 relative to the translation start site). Genes AARI_32760-32890 have a high G+C content (mean value of 69.1%, whereas the chromosome G+C content is 59.3%), probably reflecting recent horizontal transfer (see circle 12 of Figure 1, near position 3.7 Mbp).

The genome of *A. arilaitensis* encodes six putative proteins required for iron release from the Fe^3+^/siderophore complexes (AARI_02830-02840, AARI_02900, AARI_04000, AARI_05950 and AARI_32780), among these, five have no counterpart in any of the environmental *Arthrobacter* strains. *Arthrobacter aurescens* TC1, *Arthrobacter* sp. FB24 and *A. chlorophenolicus* A6 contain respectively five, three and three such proteins (COG groups 2375 and 2382). The circular representation of the *A. arilaitensis* Re117 chromosome shows that several of the regions of difference between this genome and those of the three environmental strains correspond to clusters of genes involved in siderophore biosynthesis and import that are present only in *A. arilaitensis* (near positions 0.3, 0.4, 1.2, 1.7 and 3.7 Mbp, see circle 12 of Figure 1).

**Figure 4:**
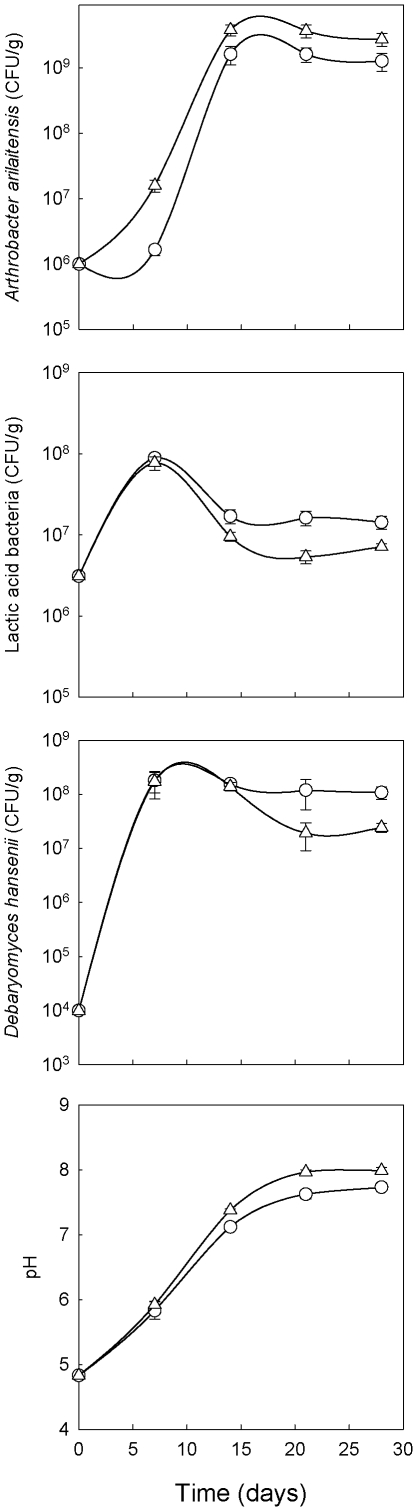
Stimulation of growth of *A. arilaitensis* in model cheeses by iron. Lactic curd was inoculated with 10^6^ cfu/g of *A. arilaitensis* Re117 and 10^4^ cfu/g the yeast *Debaryomyces hansenii* 304, without (○) or with (▵) addition of 1 mg iron per kg of curd. The values correspond to the means of three replications.

The presence in *A. arilaitensis* of a large number of genes involved in iron acquisition may be explained by a low amount of iron available at the surface of cheeses. To study this, model cheeses were produced with *A. arilaitensis* Re117, which was co-inoculated with the yeast *Debaryomyces hansenii* 304 (whose function was to increase the pH). Interestingly, addition of 1 mg/kg of iron considerably stimulated *A. arilaitensis* growth (Figure 4). At 7 days, the *A. arilaitensis* cell count was ten times higher than in the control cheeses and it remained higher by a factor of two at 14, 21 and 28 days. Growth of *D. hansenii* and of the lactic acid bacteria was not affected by the addition of iron, except that their cell count decreased more rapidly after they reached their maximum growth level.

## Discussion

Cheese is an ancient food of special importance for humanity. It is a way to conserve important constituents of milk and also a product appreciated for its organoleptic properties. The microorganisms present in all cheese varieties contribute to the typical sensory properties such as texture, taste, aroma and colour of the final product. These microorganisms are the result of centuries of adaptation of various types of yeasts, moulds and bacteria to the cheese habitat. Oxygen available at the surface of cheeses makes it possible for aerobic microorganisms to catabolize substrates such as lipids or lactic acid which are not used by the lactic acid bacteria growing within the cheeses. These catabolic activities are very important for the ripening of small soft cheeses such as smear-ripened cheeses. *Arthrobacter* species are metabolically diverse aerobic Gram-positive bacteria, widely distributed among bacterial populations in the soil [24]. Their adaptation to the cheese habitat resulted in species such as *A. arilaitensis* whose occurrence seems to be restricted to the surface of cheeses and the environment of cheese manufacturing [11]. Study of the genome of a typical *A. arilaitensis* strain is thus useful for a better understanding of the emergence of bacterial species adapted to the cheese surfaces.

The *A. arilaitensis* Re117 chromosome has large regions with high levels of synteny with the chromosomes of three environmental *Arthrobacter* strains: *A. aurescens* TC1, *A. chlorophenolicus* A6 and *Arthrobacter* sp. FB24. However, it carries a much higher load of insertion sequences than do these strains. Although about 20% of the IS were observed to have interupted *A. arilaitensis* Re117 genes, no particular functional bias was apparent. Such IS expansions have been observed in several bacterial species where it has been suggested that they are a consequence of a population bottleneck associated with a change in environmental niche [75] leading to fixation of random IS insertions in a small population. Several examples of this behaviour have been observed for pathogenic bacteria and such phenomenon might have occured in the evolution of *A. arilaitensis* Re117 from environmental strains in the limited environment of a cheese surface. Interestingly a similar phenomenon has been observed for another cheese-associated bacterium, *Lactobacillus helveticus* DPC 4571 [76].

The *A. arilaitensis* Re117 genome is smaller than that of the three other available sequenced *Arthrobacter* strains. This is due to the loss of chromosomal genes and to a lower plasmid content. A clear bias toward the loss of genes associated with catabolic activities was observed and confirmed by comparison of the capability of the *Arthrobacter* strains to grow on various carbon substrates. It is likely that this functional loss is linked to the adaptation of *A. arilaitensis* to its specialized environment, cheese surfaces. More specifically, a large decrease of ABC-type sugar import systems and of LacI-family regulators was observed. The adaptation of *A. arilaitensis* towards a more stable habitat is also illustrated by the decrease in the number of σ70-family sigma factors. As in the case of the fish pathogen *Renibacterium salmoninarum* [77], the genome sequence of *A. arilaitensis* suggests a reductive evolution from an environmental *Arthrobacter* ancestor. Interestingly, a process of genome reduction has also been suggested for the lactic acid bacterium *Lactobacillus delbrueckii* ssp. *bulgaricus* in the context of adaptation from a plant-associated habitat to the stable protein and lactose-rich milk environment [78].

*Arthrobacter* strains are well-equipped with enzymes required for the catabolism of major carbon substrates present at the cheese surface. These include: a full set of TCA cycle enzymes, a glyoxylate bypass, proteases, lipases and the enzymes for fatty acids, amino acids, and lactic acid catabolism. However, *A. arilaitensis* has several specificities which seem to be linked to its niche adaptation. One is the ability to catabolize exogenous D-galactonic acid. The presence of this compound has never been investigated in milk and in cheeses and it does not seem to be widespread in nature [79]. It is present in the urine of galactosemic patients as a result of alternative D-galactose metabolism in humans [80]. The ability of *A. arilaitensis* Re117 to catabolize D-galactonate is the consequence of a recent horizontal gene transfer of a cluster of four genes (AARI_31720-31750), whose closest orthologs are found in Gram-negative species. It is possible that in some varieties of cheeses D-galactonate may be produced transiently by oxidation from residual lactose or galactose, as described in some fungi [81]. This compound would then be utilized as a growth substrate by *Arthrobacter* species. Investigations concerning the presence of D-galactonate in cheeses and the ability of cheese microorganisms to produce this compound from lactose or galactose are needed to confirm this hypothesis.

Another specificity of *A. arilaitensis* in comparison to the three environmental *Arthrobacter* strains whose genome sequence is available, is its higher salt tolerance. Most cheeses are salted after growth of the starter lactic acid bacteria by addition of dry salt or immersion in a brine solution. One of the principal functions of salt is to control microbial growth and thereby to extend shelf-life. Consequently, microorganisms such as the surface microorganisms that grow after the salting of cheeses, are, at least transiently, under selective pressure for salt-tolerance. While several mechanisms may contribute to salt-tolerance of *A. arilaitensis*, we observed that its genome contains a higher number of glycine betaine and related osmolyte transporters, which may account for the increased salt-tolerance.

The *A. arilaitensis* genome is also well equipped with genes involved in iron acquisition. Two siderophore biosynthesis gene clusters have been identified, one having no counterpart in the three environmental *Arthrobacter* strains. In addition, 30 genes were assigned to Fe^3+^/siderophore transport components. Most had no ortholog in the environmental strains, which had between 4 and 18 of such components. It is likely that *A. arilaitensis* is able to use siderophores produced by other species sharing its habitat. Some of the genes involved in iron acquisition likely result from a recent horizontal transfer as indicated by their atypical G+C contents. Interestingly, addition of iron strongly stimulated the growth of *A. arilaitensis* Re117 in model cheeses, showing that iron acquisition is a key element for the growth of *A. arilaitensis* in cheese. Production of siderophores has been observed in strains of *Brevibacterium linens*, frequently found at the surface of smear-ripened cheeses [82]. Iron is essential in key metabolic processes such as respiration. However, cheese is a highly iron-restricted medium. Bovine milk is poor in iron (0.2–0.4 mg/l) [83], and it contains lactoferrin, a glycoprotein that has an antibacterial effect, due to its ability to chelate iron [84]. In addition, because of the presence of oxygen, iron at the surface of cheese is essentially in the oxidized ferric form, FeIII, which is extremely insoluble especially at the pH where the growth of the acido-sensitive bacterial surface flora occurs (pH>6). There may also be diffusional limitations of iron and iron-containing compounds due to the solid cheese matrix. Moreover available iron may be sequestered by yeast cells, the growth of which precedes that of surface bacteria.

It is noteworthy that lactic acid bacteria, which in cheesemaking grow at the beginning of manufacturing, are the only form of life which does not require iron. For example, certain lactobacilli contain just one or two iron atoms per cell, levels considered too low to provide iron with any conceivable biological function [85], [86]. They possess unique cofactors that do not require iron and this feature may explain their ability to grow in iron-restricted environments such as milk and the natural gut flora of breast-fed infants [87]. However, important substrates at the surface of cheeses such as lipids, amino acids or lactic acid, which are hardly used by the fermentative lactic acid bacteria, can be catabolized by microorganisms with iron-requiring respiratory activity. The lactic acid bacteria are thus well-adapted to the inner-cheese environment but at the surface there is likely a selective advantage for aerobic strains with efficient iron acquisition systems.

Availability of iron is probably a key factor influencing growth of microorganisms at the cheese surface. Consequently, investigation of the role of iron on the microbial ecology of the cheese surface microflora may be useful for a better growth control of the desirable microorganisms as well as the spoilage microorganisms and pathogens. For example, it would be interesting to determine whether the high incidence of *Listeria monocytogenes* at the surface of smear-ripened cheses [88] has a link with its ability to use a variety of exogenous siderophores [89]. In addition, this species is known for its high salt resistance [90], which may also favour its growth in cheese. The metabolism of iron by the microorganisms of cheese may also have implications in terms of human health. Indeed, fungal siderophores have been detected in several varieties of cheese [91] and recently it has been shown that such siderophores function as protective agents of low density lipoprotein (LDL) oxidation and are promising anti-atherosclerotic metabolites [92]. The authors of this study speculated that the consumption of traditional mould-ripened food products such as aged cheeses may lower the risk of cardiovascular disease.

Volatile sulfur compounds are major flavour compounds in many types of cheeses. However, no evidence could be found for the presence, in *A. arilaitenisis* Re117, of enzymes releasing methane thiol, the precursor of these compounds. This is consistent with a previous study, in which no significant production of volatile sulfur compounds was observed for *Arthrobacter* strains growing in liquid media [93]. In contrast, the presence several lipases, including a secretory lipase, indicates that *A. arilaitensis* may contribute to the flavour of cheese by producing fatty acids and their degradation compounds.

The yellow pigmentation of *A. arilaitensis* Re117 colonies is probably due to the presence of a cluster of genes for carotenoid biosynthesis. It has been shown that carotenoids from yellow-pigmented bacteria contribute to the coloration of smear-ripened cheeses which are characterised by the presence of an orange-brown, sticky surface [61]. Cheese colour is an important criterion of acceptance by cheese consumers, especially for smear-ripened cheeses. However, cheese rind coloration is a complex process involving physical and chemical factors as well as biotic interactions [61], [94]. To better understand the key factors involved in colour generation by the bacteria from the cheese surface, it would be interesting to quantify the expression of the carotenoid biosynthesis genes such as those from *A. arilaitensis*. Quantification of gene expression in cheeses would also be interesting to determine whether genes involved in useful properties, such as those encoding for biogenic amine catabolic enzymes, are expressed during cheese manufacturing.

In conclusion, the comparison of the genome of *A. arilaitensis* Re117 with the genomes of environmental *Arthrobacter* strains provides evidence that adaptation to an environment created by man, the cheese surface, resulted in Insertion Sequence element expansion and the loss of genes associated with catabolic activities. We propose that in this habitat, there is a strong selective pressure for strains with efficient iron acquisition and salt-tolerance systems, together with abilities to catabolize substrates such as lactic acid, lipids and amino acids. More studies are needed to evaluate the importance of microbial interactions, such as the ability to use siderophores produced by the microorganisms sharing the cheese habitat or the production and consumption of compounds such as D-galactonate. This may provide new ideas for a better control of the growth of ripening microorganisms and for reducing the occurrence of spoilage microorganisms and pathogens. Our analysis of the genome also provides genetic data for investigating the generation of interesting functional properties such as colour and aroma compound production.

## Supporting Information

Figure S1 Taxonomical distribution of *Arthrobacter arilaitensis* Re117 CDSs in other bacterial genomes. A CDS is considered to have a cognate present in the compared genome if its BLAST best hit presents an e-value lower than 10^-3^ with an overlap higher than 80%. The subset of organisms shown includes those for which >20 best matches were seen. Transposases have been excluded from the analysis.
(PDF)

Figure S2 Venn diagram representing the shared genes between the four sequenced *Arthrobacter* strains. Genes are considered as shared if they are orthologous (see section "Genome analysis and annotation").Transposases have been excluded from the analysis.
(PDF)

Figure S3 Functional categories of *Arthrobacter* genes using the cluster of orthologous group scheme. The genes present in the three environmental strains but absent in *A. arilaitensis* were also analyzed (designated as "shared TC1-FB24-A6"). Functional assignments were used according to the COG database (http://www.ncbi.nlm.nih.gov/COG/) and the transposases were excluded from analysis. C, energy production; D, cell division; E, amino acid metabolism; F, nucleotide metabolism; G, carbohydrate metabolism; H, coenzyme metabolism; I, lipid metabolism; J, translation; K, transcription; L, DNA replication, recombination and repair; M, cell wall/membrane biogenesis; N, cell motility; O, post-translational modification; P, inorganic ion metabolism; Q, secondary metabolite biosynthesis, transport and catabolism; R, general function prediction only; S, function unknown; T, signal transduction; U, intracellular trafficking and secretion; V, defense mechanism.
(PDF)

Figure S4 Synteny between the *A. arilaitensis* Re117 chromosome and the chromosomes of *A. aurescens* TC1, *Arthrobacter* sp. FB24 and *A. chlorophenolicus* A6. The graphic shows X-Y plots of dots forming syntenic regions between the chromosomes. Each dot represents a predicted *A. arilaitensis* protein having an ortholog in another *Arthrobacter* chromosome with coordinates corresponding to the position of the respective coding region in each genome. The orthologs were identified as described in section "Genome analysis and annotation". Red dots correspond to CDSs in the same orientation and blue dots to reverse orientation.
(PDF)

Figure S5 Circular representation of the chromosomes of *A. aurescens* TC1, *Arthrobacter* sp. FB24 and *A. chlorophenolicus* A6 showing the orthology relations with *A. arilaitensis* Re117. The outermost circle (circle 1) represents the scale in Mbp. Circles 2 and 3 represent CDSs on positive (red) and negative (blue) strands. Circle 4 represents CDSs (in orange) with an ortholog in *A. arilaitensis* Re117.
(PDF)

Figure S6 Effect of salt concentration on the growth rate of *Arthrobacter* strains. The strains were cultivated in brain heart infusion broth at 25°C and in aerobic conditions. The maximum growth rate was determined by the slope of the plot relating ln absorbance (600 nm) versus time.
(PDF)

Table S1 Codon usage and codons covered by the 64 transfer RNAs of *A. arilaitensis* Re117.
(DOC)

Table S2 Pseudogenes found in the *A. arilaitensis* Re117 genome.
(DOC)

Table S3 Genes in the *A. arilaitensis* Re117 genome involved in repair of DNA lesions.
10.1371/journal.pone.0015489.s010
(DOC)

Table S4 Predicted transporters in *Arthrobacter arilaitensis* Re117.
(DOC)

Table S5 Copy number and distribution of IS families in four *Arthrobacter* species.
(XLS)

Table S6 Insertion sequences identified in the genome of *A. arilaitensis.*
(XLS)

Table S7 Predicted transcriptional regulatory proteins of *A. arilaitensis* Re117.
(DOC)

Table S8 Catabolism of amino acids by *A. arilaitensis* Re117.
(DOC)

Table S9 Utilization of various carbon sources by *Arthrobacter* strains growing aerobically on Biotype 100 strips.
(DOC)

Table S10 Occurence of COG hits related to sugar ABC-type transport systems.
(DOC)

Table S11 Genes in the *A. arilaitensis* Re117 genome with putative function in protein degradation.
(DOC)

Table S12 Genes assigned to glycine betaine and related osmolyte transporters in the *A. arilaitensis* genome.
(DOC)

Table S13 Predicted lipase and esterase genes in *A. arilaitensis* Re117.
(DOC)

Table S14 Predicted acyl-CoA dehydrogenase and fatty acid--CoA ligase genes in *A. arilaitensis* Re117.
(DOC)

Table S15 Distribution of ABC families in *Arthrobacter arilaitensis* Re117.
(DOC)
