# Supplementary figures and images for: The Arthrobacter arilaitensis Re117 Genome Sequence Reveals Its Genetic Adaptation to the Surface of Cheese
Source: PLoS One. 2010 Nov 24;5(11):e15489. doi: 10.1371/journal.pone.0015489 (PMC2991359; doi:10.1371/journal.pone.0015489)

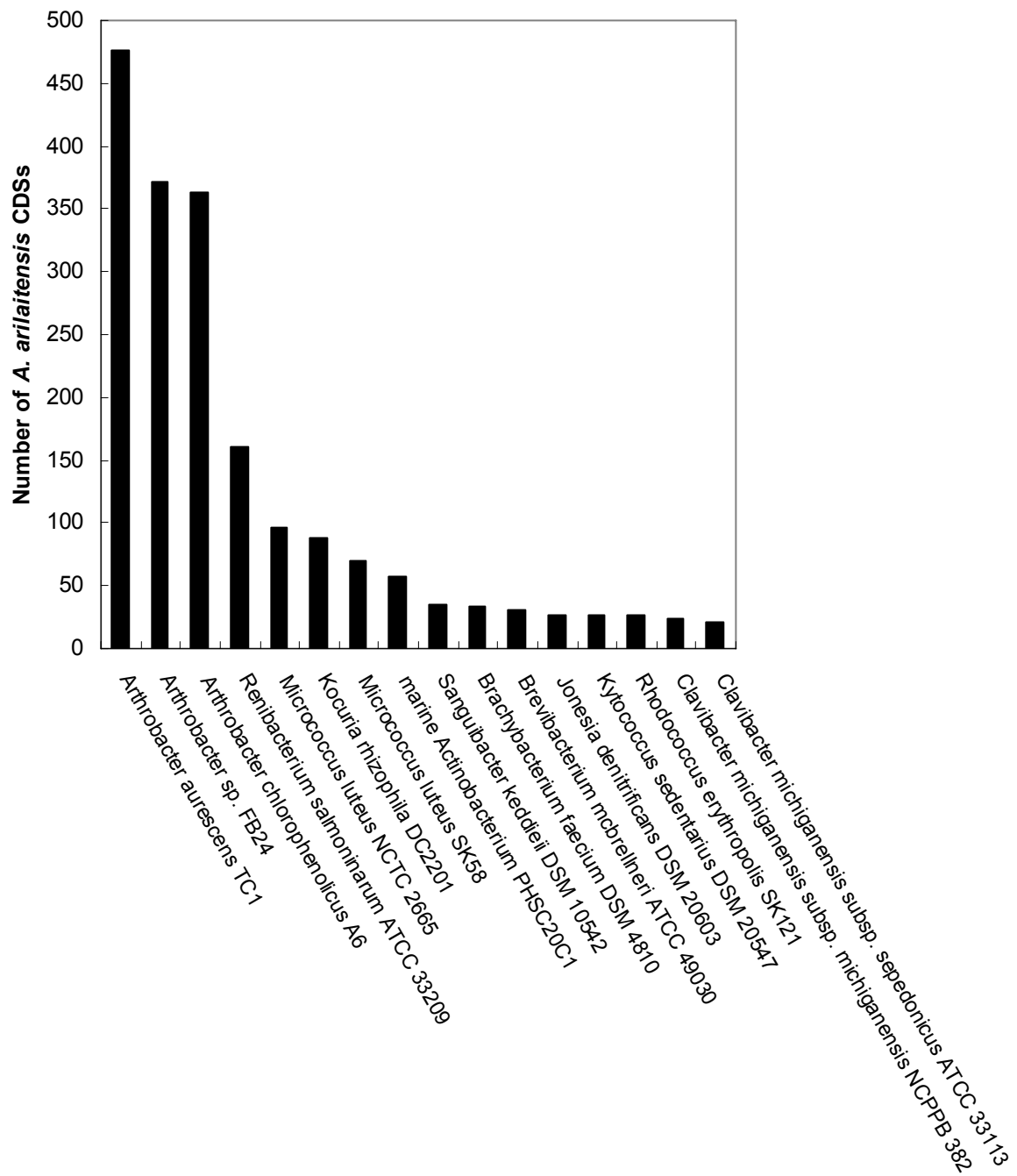

Supplement: Figure S1 — Taxonomical distribution of Arthrobacter arilaitensis Re117 CDSs in other bacterial genomes. A CDS is considered to have a cognate present in the compared genome if its BLAST best hit presents an e-value lower than 10-3 with an overlap higher than 80%. The subset of organisms shown includes those for which >20 best matches were seen. Transposases have been excluded from the analysis. (PDF) [file pone.0015489.s001.pdf]

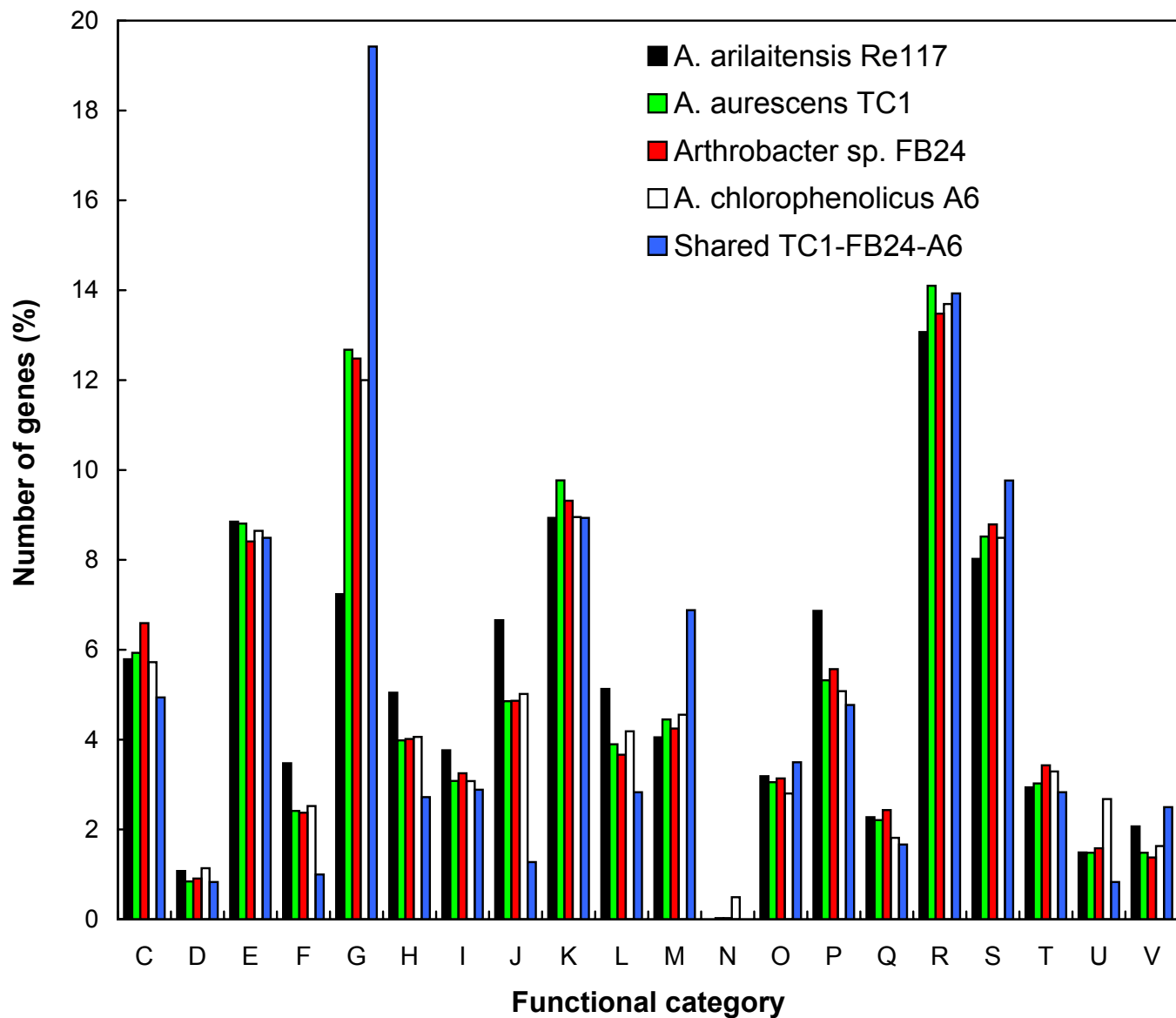

Supplement: Figure S3 — Functional categories of Arthrobacter genes using the cluster of orthologous group scheme. The genes present in the three environmental strains but absent in A. arilaitensis were also analyzed (designated as "shared TC1-FB24-A6"). Functional assignments were used according to the COG database (http://www.ncbi.nlm.nih.gov/COG/) and the transposases were excluded from analysis. C, energy production; D, cell division; E, amino acid metabolism; F, nucleotide metabolism; G, carbohydrate metabolism; H, coenzyme metabolism; I, lipid metabolism; J, translation; K, transcription; L, DNA replication, recombination and repair; M, cell wall/membrane biogenesis; N, cell motility; O, post-translational modification; P, inorganic ion metabolism; Q, secondary metabolite biosynthesis, transport and catabolism; R, general function prediction only; S, function unknown; T, signal transduction; U, intracellular trafficking and secretion; V, defense mechanism. (PDF) [file pone.0015489.s003.pdf]

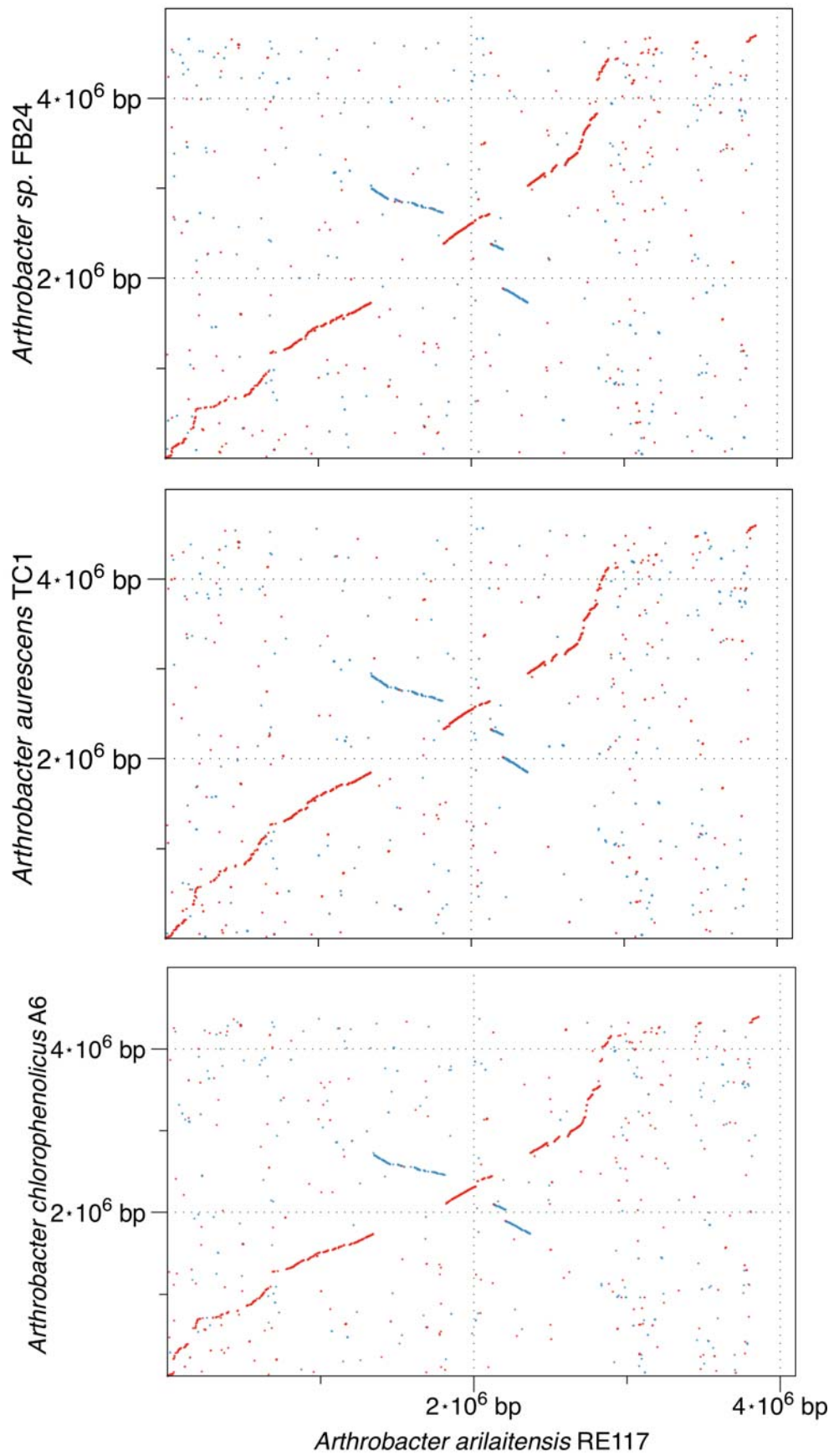

Supplement: Figure S4 — Synteny between the A. arilaitensis Re117 chromosome and the chromosomes of A. aurescens TC1, Arthrobacter sp. FB24 and A. chlorophenolicus A6. The graphic shows X-Y plots of dots forming syntenic regions between the chromosomes. Each dot represents a predicted A. arilaitensis protein having an ortholog in another Arthrobacter chromosome with coordinates corresponding to the position of the respective coding region in each genome. The orthologs were identified as described in section "Genome analysis and annotation". Red dots correspond to CDSs in the same orientation and blue dots to reverse orientation. (PDF) [file pone.0015489.s004.pdf]

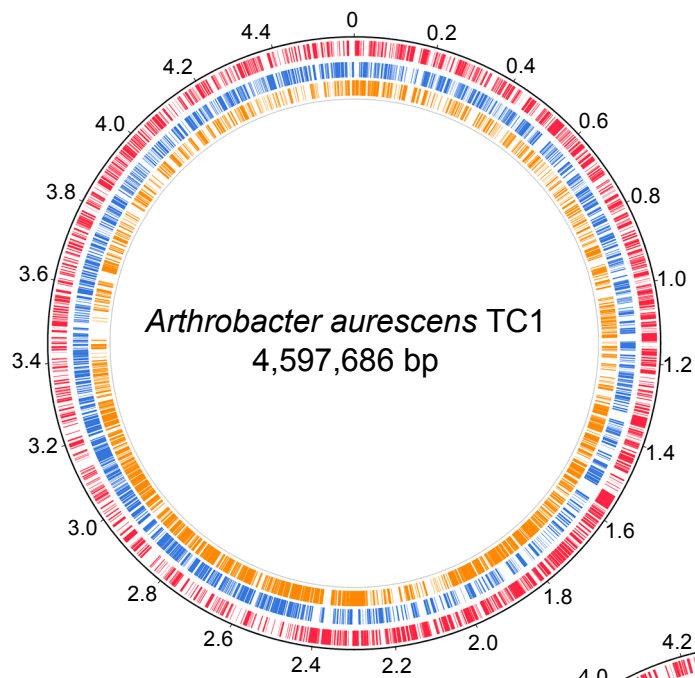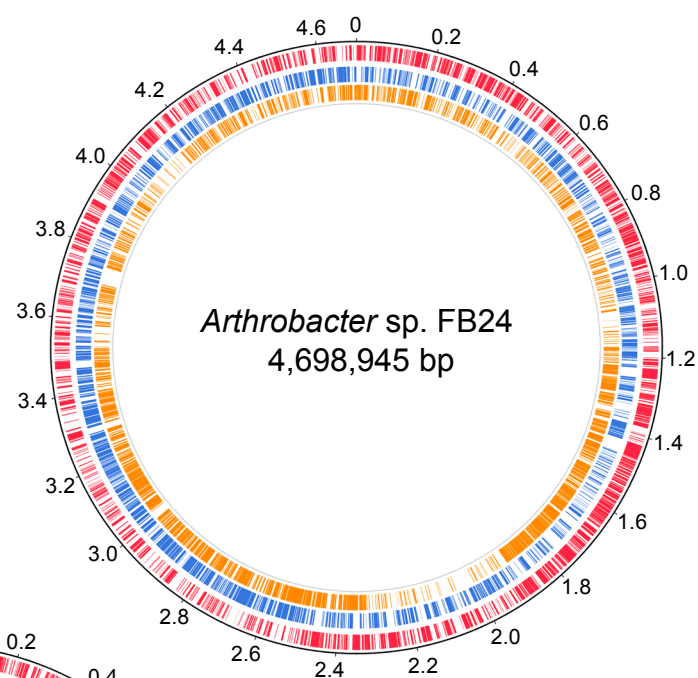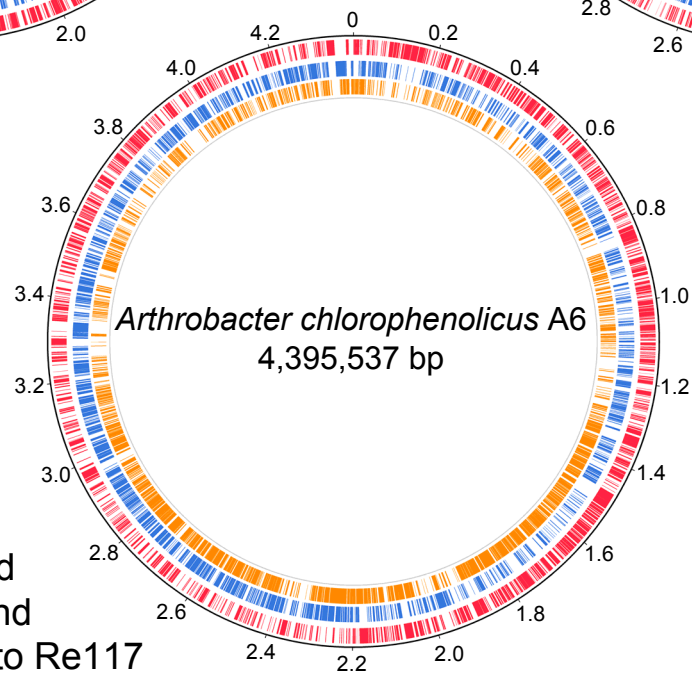

red = CDS on forward strand  
blue = CDS on reverse strand  
orange = CDS orthologous to Re117

Supplement: Figure S5 — Circular representation of the chromosomes of A. aurescens TC1, Arthrobacter sp. FB24 and A. chlorophenolicus A6 showing the orthology relations with A. arilaitensis Re117. The outermost circle (circle 1) represents the scale in Mbp. Circles 2 and 3 represent CDSs on positive (red) and negative (blue) strands. Circle 4 represents CDSs (in orange) with an ortholog in A. arilaitensis Re117. (PDF) [file pone.0015489.s005.pdf]

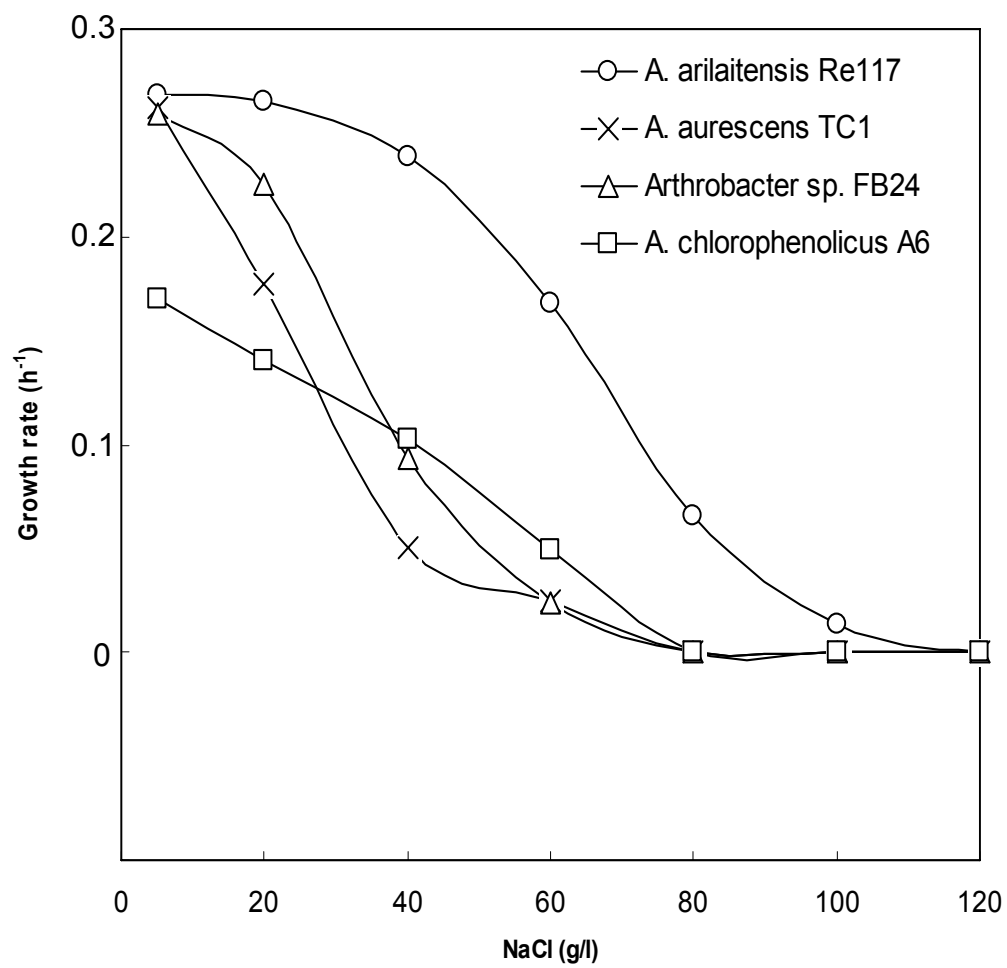

Supplement: Figure S6 — Effect of salt concentration on the growth rate of Arthrobacter strains. The strains were cultivated in brain heart infusion broth at 25°C and in aerobic conditions. The maximum growth rate was determined by the slope of the plot relating ln absorbance (600 nm) versus time. (PDF) [file pone.0015489.s006.pdf]
