# Supplementary material for: The Arthrobacter arilaitensis Re117 Genome Sequence Reveals Its Genetic Adaptation to the Surface of Cheese
Source: PLoS One. 2010 Nov 24;5(11):e15489. doi: 10.1371/journal.pone.0015489 (PMC2991359; doi:10.1371/journal.pone.0015489)
Supplement: Figure S2 — Venn diagram representing the shared genes between the four sequenced Arthrobacter strains. Genes are considered as shared if they are orthologous (see section "Genome analysis and annotation").Transposases have been excluded from the analysis. (PDF) [file pone.0015489.s002.pdf]

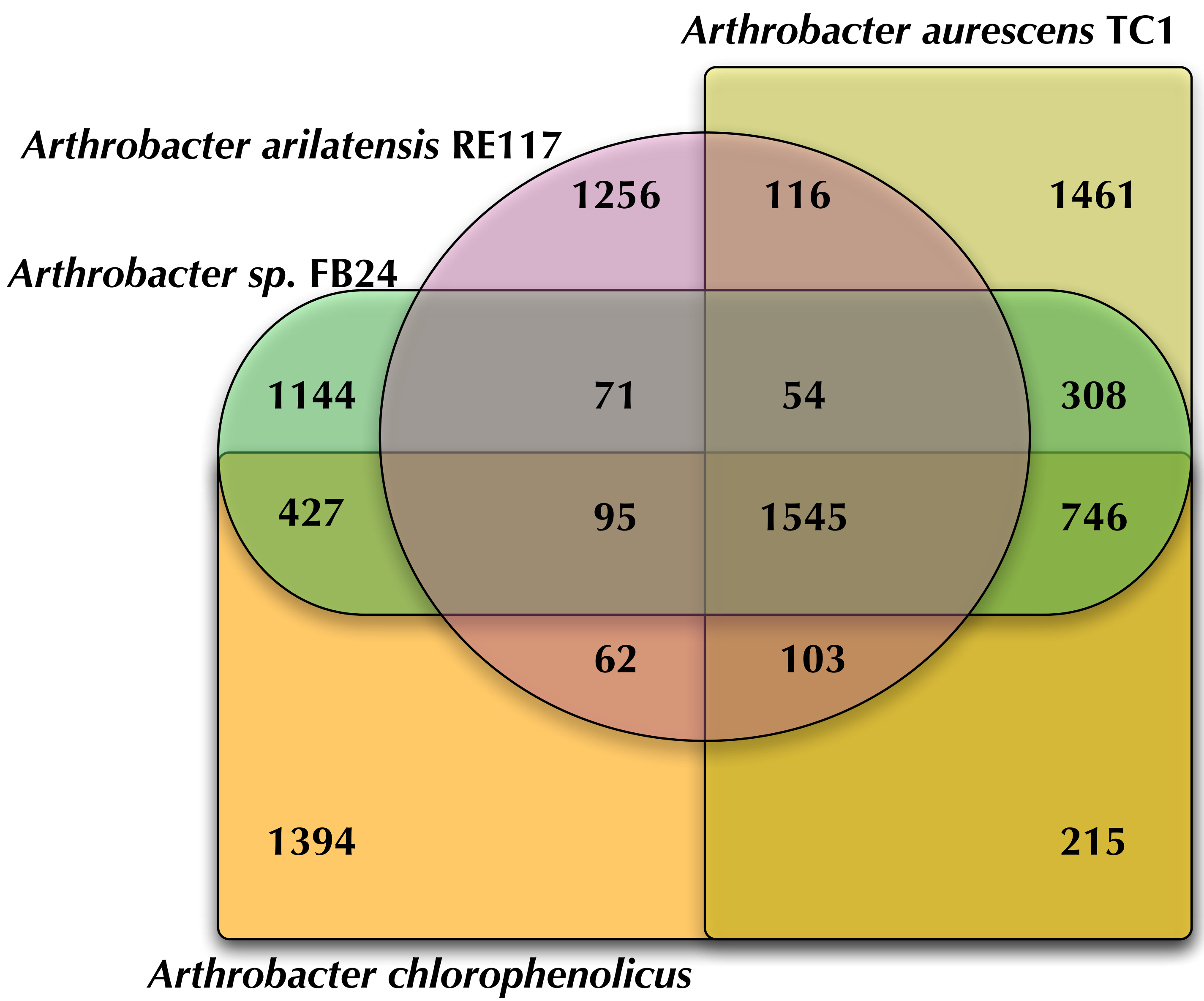
Arthrobacter aurescens TC1
Arthrobacter arilatensis RE117
Arthrobacter sp. FB24
Arthrobacter chlorophenolicus
1256
116
1461
1144
71
54
308
427
95
1545
746
62
103
1394
215
