# Supplementary material for: The Arthrobacter arilaitensis Re117 Genome Sequence Reveals Its Genetic Adaptation to the Surface of Cheese
Source: PLoS One. 2010 Nov 24;5(11):e15489. doi: 10.1371/journal.pone.0015489 (PMC2991359; doi:10.1371/journal.pone.0015489)
Supplement: Table S1 — Codon usage and codons covered by the 64 transfer RNAs of A. arilaitensis Re117. (DOC) [file pone.0015489.s007.doc]

**Table S1** Codon usage and codons covered by the 64 transfer RNAs of *A. arilaitensis* Re117.^a^

|  | **T** | **C** | **A** | **G** |  |
| --- | --- | --- | --- | --- | --- |
| **T** | TTT(Phe)0.158 | TCT(Ser)0.070 | TAT(Tyr)0.267 | TGT(Cys)0.133 | **T** |
|  | TTC(Phe)0.842 [1] | TCC(Ser)0.304 [1] | TAC(Tyr)0.733 [1] | TGC(Cys)0.867 [1] | **C** |
|  | TTA(Leu)0.017 [1] | TCA(Ser)0.084 [1] | TAA(Stop)0.305 | TGA(Stop)0.283 | **A** |
|  | TTG(Leu)0.183 [1] | TCG(Ser)0.254 [1] | TAG(Stop)0.412 | TGG(Trp)1.000 [1] | **G** |
| **C** | CTT(Leu)0.090 | CCT(Pro)0.144 | CAT(His)0.358 | CGT(Arg)0.182 [2] | **T** |
|  | CTC(Leu)0.165 [1] | CCC(Pro)0.198 [1] | CAC(His)0.642 [2] | CGC(Arg)0.558 | **C** |
|  | CTA(Leu)0.037 [1] | CCA(Pro)0.219 [3] | CAA(Gln)0.246 [1] | CGA(Arg)0.088 | **A** |
|  | CTG(Leu)0.508 [1] | CCG(Pro)0.439 [1] | CAG(Gln)0.754 [2] | CGG(Arg)0.172 [1] | **G** |
| **A** | ATT(Ile)0.250 | ACT(Thr)0.125 | AAT(Asn)0.307 | AGT(Ser)0.050 | **T** |
|  | ATC(Ile)0.720 [1] | ACC(Thr)0.653 [2] | AAC(Asn)0.693 [2] | AGC(Ser)0.238 [1] | **C** |
|  | ATA(Ile)0.030 | ACA(Thr)0.062 [1] | AAA(Lys)0.221 [1] | AGA(Arg)0.338 [1] | **A** |
|  | ATG(Met)1.000 [5] | ACG(Thr)0.160 [1] | AAG(Lys)0.779 [3] | AGG(Arg)0.662 [1] | **G** |
| **G** | GTT(Val)0.176 | GCT(Ala)0.161 | GAT(Asp)0.451 | GGT(Gly)0.168 | **T** |
|  | GTC(Val)0.328 [2] | GCC(Ala)0.435 [2] | GAC(Asp)0.549 [2] | GGC(Gly)0.523 [4] | **C** |
|  | GTA(Val)0.103 [2] | GCA(Ala)0.184 [2] | GAA(Glu)0.573 [1] | GGA(Gly)0.167 [1] | **A** |
|  | GTG(Val)0.393 [1] | GCG(Ala)0.220 | GAG(Glu)0.427 [2] | GGG(Gly)0.141 [1] | **G** |

^a^The codons presumably recognized by a tRNA with a wobble base pair are highlighted. The numbers 0.0 to 1.0 indicate the proportion of the amino acids encoded by a given codon. The number of genes corresponding to each codon is indicated in brackets.
