# Supplementary material for: The Arthrobacter arilaitensis Re117 Genome Sequence Reveals Its Genetic Adaptation to the Surface of Cheese
Source: PLoS One. 2010 Nov 24;5(11):e15489. doi: 10.1371/journal.pone.0015489 (PMC2991359; doi:10.1371/journal.pone.0015489)
Supplement: Table S2 — Pseudogenes found in the A. arilaitensis Re117 genome. (DOC) [file pone.0015489.s008.doc]

**Table S2** Pseudogenes found in the *A. arilaitensis* Re117 genome.^a^

| **Locus tag AARI_** | **Product** | **Inactivation by** |
| --- | --- | --- |
|  |  |  |
| Chromosome |  |  |
| 00300, 00310 | MerR-family transcriptional regulator | frameshift |
| 00650 | protein of unknown function | truncation |
| 00740, 00750 | protein of unknown function | frameshift |
| 01270 | MFS superfamily transporter | insertion sequence |
| 01510, 01520 | protein of unknown function | frameshift |
| 01590 | glycosyl hydrolase | truncation |
| 02250 | sodium/solute symporter | truncation |
| 02270, 02280 | reverse transcriptase/maturase | insertion sequence |
| 02310 | polysaccharide deacetylase | truncation |
| 02340 | fructose-specific phosphotransferase system IIABC components | truncation |
| 02400 | TraG-family protein | truncation |
| 02700 | CoA-binding domain protein | truncation |
| 03070, 03080 | CitA signal transduction histidine kinase | frameshift |
| 04060, 04100 | AraC-family transcriptional regulator | insertion sequence |
| 04330, 04340 | transcriptional regulator | in-frame stop |
| 04770, 04780 | respiratory nitrate reductase subunit alpha | frameshift |
| 04950, 04960 | sugar ABC transporter, substrate-binding protein | frameshift |
| 05010 | GNAT-family acetyltransferase | truncation |
| 06590 | short-chain dehydrogenase/reductase | truncation |
| 06600, 06610 | protein of unknown function | frameshift |
| 06620 | protein of unknown function | truncation |
| 06630 | protein of unknown function | truncation |
| 03730 | restriction-modification system endonuclease | truncation |
| 06870 | TraG-family protein | truncation |
| 06880 | GNAT-family acetyltransferase | truncation |
| 07020 | protein of unknown function | truncation |
| 07060 | FAD-dependent pyridine nucleotide-disulphide oxidoreductase | truncation |
| 07590, 07600 | renal dipeptidase family protein | frameshift |
| 08030 | DNA gyrase subunit | insertion sequence |
| 08050 | resolvase | insertion sequence |
| 08280, 08290 | NAD dependent epimerase/dehydratase family protein | in-frame stop |
| 08380, 08390 | dicarboxylate/amino acid transporter | insertion sequence |
| 08480, 08490 | transglutaminase-like protease | frameshift |
| 08670, 08680 | glucosamine-6-phosphate isomerase | frameshift |
| 08790 | protein of unknown function | truncation |
| 08800 | GNAT-family acetyltransferase | truncation |
| 08830 | protein of unknown function | truncation |
| 09000 | peptidase | truncation |
| 09400, 09410 | glycerate kinase | frameshift |
| 10220 | protein of unknown function | truncation |
| 10370, 10380, 10390 | MFS superfamily transporter | in-frame stops |
| 10580 | protein of unknown function | truncation |
| 10680, 10690, 10700 | short chain fatty acid transporter | in-frame stops |
| 11780 | cytidine deaminase | truncation |
| 11800, 11810 | protein of unknown function | frameshift |
| 12510 | hydrolase | truncation |
| 12950 | protein of unknown function | truncation |
| 13240 | MFS superfamily transporter | insertion sequence |
| 13260, 13270 | protein of unknown function | frameshift |
| 13350, 13370 | peroxidase | insertion sequence |
| 13370 | glycosyl hydrolase | insertion sequence |
| 13610 | OsmC-like protein | insertion sequence |
| 14100 | protein of unknown function | insertion sequence |
| 14580, 14592 | family 2 glycosyl transferase | insertion sequence, partial duplication |
| 14990, 15000 | protein of unknown function | in-frame stop |
| 15730 | dehydrogenase | truncation |
| 15740, 15750, 15760 | protein of unknown function | in-frame stops |
| 16050 | N-acetylmannosamine-6-phosphate epimerase | truncation |
| 16080, 16090 | protein of unknown function | frameshift |
| 16240, 16250 | protein of unknown function | frameshift |
| 16240 | protein of unknown function | truncation |
| 17580 | transcriptional regulator | truncation |
| 17960 | transporter | truncation |
| 18980, 18990 | glycogen debranching enzyme | in-frame stop |
| 19710, 19720 | benzoate permease | in-frame stop |
| 20300 | serine/threonine protein kinase | insertion sequence |
| 20380 | transcriptional regulator | truncation |
| 22730, 22740, 22750 | metallophosphoesterase | insertion sequence |
| 22760 | carboxylate-amine ligase | truncation |
| 22780 | reverse transcriptase/maturase related protein | insertion sequence |
| 22870 | protein of unknown function | truncation |
| 22980, 22990 | metallophosphoesterase | in-frame stop |
| 23420 | protein of unknown function | truncation |
| 24860 | protein of unknown function | truncation |
| 24870 | protein of unknown function | truncation |
| 24880 | protein of unknown function | truncation |
| 25250 | protein of unknown function | truncation |
| 25350, 25360, 25370 | glycosyl hydrolase, family 13 | frameshifts, partial duplication |
| 26780, 26790 | glutamate synthase | insertion sequence |
| 26970, 26980 | AsnC/Lrp-family transcriptional regulator | insertion sequence |
| 27420 | sugar ABC transporter permease protein | truncation |
| 27580, 27590 | ribose ABC transporter substrate-binding protein | frameshift |
| 27680, 27690 | 4-hydroxy-2-oxovalerate aldolase | frameshift |
| 27720 | flavoprotein monooxygenase | truncation |
| 28380 | sugar ABC transporter permease protein | truncation |
| 28390 | NAD-dependent aldehyde dehydrogenase | truncation |
| 28400, 28410, 28420 | Zn-dependent alcohol dehydrogenase | frameshifts |
| 28550, 28560 | transcriptional regulator | frameshift |
| 28610, 28620 | L-asparagine permease | in-frame stop |
| 28830, 28840 | cytidine/deoxycytidylate deaminase | frameshift |
| 28940, 28950 | uracil/xanthine permease | frameshift |
| 29020 | ABC transporter permease protein | truncation |
| 29200, 29210, 29220, 29230 | protein of unknown function | frameshifts, in-frame stops |
| 29240 | protein of unknown function | insertion sequence |
| 29460 | ATP/GTP-binding protein | insertion sequence |
| 29510 | protein of unknown function | truncation |
| 29540, 29590, 29600, 29620 | TraA-like conjugal transfer protein | insertion sequence, partial duplication |
| 29690 | resolvase | truncation |
| 29730 | endonuclease | truncation |
| 30040, 30050 | protein of unknown function | frameshift |
| 30490 | metal-dependent transcriptional regulator | truncation |
| 30630, 30640 | alcohol dehydrogenase family protein | frameshift |
| 30770, 30780 | GNAT-family acetyltransferase | frameshift |
| 30980 | protein of unknown function | truncation |
| 31160, 31170 | metal-dependent amidase/aminoacylase/carboxypeptidase | frameshift |
| 31310 | Ku domain-containing protein | truncation |
| 31320 | ATP dependent DNA ligase | truncation |
| 32050 | protein of unknown function | insertion sequence |
| 32200 | glycine betaine transporter | truncation |
| 32210 | MFS superfamily transporter | truncation |
| 32580 | MFS superfamily transporter | truncation |
| 32610 | MFS superfamily transporter | truncation |
| 32700 | MFS superfamily transporter | truncation |
| 32710 | MFS superfamily transporter | truncation |
| 32940 | 4-hydroxyphenylacetate 3-hydroxylase family protein | truncation |
| 33010, 33020 | protein of unknown function | frameshift |
| 33130, 33140, 33150 | benzoate 1,2-dioxygenase subunit | frameshifts |
| 33170 | protein of unknown function | truncation |
| 33240 | protein of unknown function | truncation |
| 33380, 33390 | protein of unknown function | frameshift |
| 33500, 33510 | protein of unknown function | frameshift |
| 33790 | protein of unknown function | truncation |
| 33860 | protein of unknown function | truncation |
|  |  |  |
| Plasmid pRE117-1 | |  |
| AARI_pI00020 | plasmid partitioning protein | truncation |
| AARI_pI00370 | protein of unknown function | truncation |
|  |  |  |
| Plasmid pRE117-2 | |  |
| / | |  |

^a^Pseudogenes related to insertion sequences are not listed.
