# Supplementary material for: The Arthrobacter arilaitensis Re117 Genome Sequence Reveals Its Genetic Adaptation to the Surface of Cheese
Source: PLoS One. 2010 Nov 24;5(11):e15489. doi: 10.1371/journal.pone.0015489 (PMC2991359; doi:10.1371/journal.pone.0015489)
Supplement: Table S3 — Genes in the A. arilaitensis Re117 genome involved in repair of DNA lesions. 10.1371/journal.pone.0015489.s010 (DOC) [file pone.0015489.s009.doc]

**Table S3** Genes in the *A. arilaitensis* Re117 genome involved in repair of DNA lesions.*^a^*

| **Locus tag**  **AARI_** | **Gene symbol** | **EC number** | **Predicted protein product** |
| --- | --- | --- | --- |
|  |  |  |  |
| Chromosome | |  |  |
| 01100 | *mutY* | 3.2.2.- | putative A/G-specific DNA glycosylase |
| 01120 | *disA* |  | DNA integrity scanning protein DisA |
| 01130 | *radA* |  | DNA repair protein RadA |
| 01400 | *uveA* | 3.2.2.- | pyrimidine dimer DNA glycosylase |
| 01870 | *xthA* | 3.1.11.2 | exodeoxyribonuclease III |
| 02490 | *ogt* | 2.1.1.63 | methylated-DNA--[protein]-cysteine S-methyltransferase |
| 03170*^b^* | *recR* |  | recombination protein RecR |
| 03500 | *umuD* | 2.7.7.7 | putative DNA polymerase subunit UmuD |
| 03510 | *umuC* | 2.7.7.7 | putative DNA polymerase subunit UmuC |
| 04220 | *nei* | 3.2.2.- | DNA glycosylase |
| 04230 |  | 3.6.1.- | putative ATP-dependent DNA helicase |
| 05490 | *ung* | 3.2.2.- | uracil-DNA glycosylase |
| 05960 | *alkA* | 3.2.2.21 | putative DNA-3-methyladenine glycosylase II |
| 05970 | *tagA* | 3.2.2.20 | putative DNA-3-methyladenine glycosylase I |
| 07150 |  | 3.1.11.2 | exodeoxyribonuclease III |
| 08170 | *mfd* | 3.6.1.- | transcription-repair-coupling factor |
| 11011 | *recA* |  | RecA bacterial DNA recombination protein |
| 11020*^b^* | *recX* |  | putative regulatory protein RecX |
| 11620 | *recN* |  | DNA repair protein RecN |
| 14440 | *recG* | 3.6.1.- | putative ATP-dependent DNA helicase RecG |
| 14640 | *mutM* | 3.2.2.23/ 4.2.99.18 | formamidopyrimidine-DNA glycosylase |
| 14690 |  | 3.2.2.- | putative 3-methyladenine DNA glycosylase |
| 15590 | *uvrA* |  | excinuclease ABC subunit A |
| 16780 | *recO* |  | DNA repair protein RecO |
| 17460 | *ruvB* | 3.6.1.- | Holliday junction ATP-dependent DNA helicase RuvB |
| 17470 | *ruvA* | 3.6.1.- | Holliday junction ATP-dependent DNA helicase RuvA |
| 17480 | *ruvC* | 3.1.22.4 | crossover junction endodeoxyribonuclease RuvC |
| 18540 |  | 3.2.2.- | DNA glycosylase |
| 18680 | *uvrC* |  | excinuclease ABC subunit C |
| 19110 | *uvrA* |  | excinuclease ABC subunit A |
| 20850 | *dinB* | 2.7.7.7 | putative DNA polymerase IV |
| 21380 |  | 2.1.1.63 | putative methylated-DNA--[protein]-cysteine S-methyltransferase |
| 22390 | *xseA* | 3.1.11.6 | exodeoxyribonuclease VII large subunit |
| 22400 | *xseB* | 3.1.11.6 | exodeoxyribonuclease VII small subunit |
| 25000 | *nth* | 4.2.99.18 | DNA-(apurinic or apyrimidinic site) lyase |
| 27520 | *uvrA* |  | excinuclease ABC subunit A |
| 31360 |  | 4.1.99.3 | deoxyribodipyrimidine photo-lyase |
| 31400 | *vsr* | 3.1.-.- | putative very short patch repair endonuclease |
|  |  |  |  |
| Plasmid pRE117-1 | | |  |
| AARI_pI00070 |  | 2.7.7.7 | UmuD-like protein |
| AARI_pI00080 |  | 2.7.7.7 | UmuC-like protein |

^a^The *A. arilaitensis* genes having no ortholog in *A. aurescens* TC1, *A. chlorophenolicus* A6 and *Arthrobacter* sp. FB24 are underlined.

^b^Orthologs present in the environmental *Arthrobacter* strains but the predicted *A. arilaitensis* gene size is higher of about 200 nucleotides.
