# Supplementary material for: The Arthrobacter arilaitensis Re117 Genome Sequence Reveals Its Genetic Adaptation to the Surface of Cheese
Source: PLoS One. 2010 Nov 24;5(11):e15489. doi: 10.1371/journal.pone.0015489 (PMC2991359; doi:10.1371/journal.pone.0015489)
Supplement: Table S4 — Predicted transporters in Arthrobacter arilaitensis Re117. (DOC) [file pone.0015489.s010.doc]

**Table S4** Predicted transporters in *Arthrobacter arilaitensis* Re117.^a^

|  |  |  | Specific for *A. arilaitensis*^b^ | |  |
| --- | --- | --- | --- | --- | --- |
| TC nomenclature | Family | Number | Number | Percent | putative substrates |
| 1. Channels/Pores | | 5 | 0 | 0.0 |  |
| 1.A.8 | MIP family | 1 | 0 | 0.0 | various |
| 1.A.11 | Amt family | 1 | 0 | 0.0 | ammonia |
| 1.A.22 | MscL family | 1 | 0 | 0.0 | osmolytes, ions |
| 1.A.23 | MscS family | 1 | 0 | 0.0 | osmolytes, ions |
| 1.A.35 | MIT family | 1 | 0 | 0.0 | divalent metal ions |
|  |  |  |  |  |  |
| 2. Electrochemical potential-driven transporters | | 137 | 35 | 25.5 |  |
| 2.A.1 | MFS superfamily | 63 | 19 | 30.2 | various |
| 2.A.1.1 | SP family | 1 | 0 | 0.0 |  |
| 2.A.1.2 | DHA1 family | 2 | 0 | 0.0 |  |
| 2.A.1.3 | DHA2 family | 8 | 3 | 37.5 |  |
| 2.A.1.5 | OHS family | 1 | 1 | 100.0 |  |
| 2.A.1.6 | MHS family | 10 | 1 | 10.0 |  |
| 2.A.1.8 | NNP family | 2 | 0 | 0.0 |  |
| 2.A.1.14 | ACS family | 1 | 0 | 0.0 |  |
| 2.A.1.15 | AAHS family | 1 | 0 | 0.0 |  |
| 2.A.1.18 | PP family | 1 | 1 | 100.0 |  |
| 2.A.1.21 | DHA3 family | 2 | 0 | 0.0 |  |
| 2.A.1.38 | EntS family | 1 | 0 | 0.0 |  |
| 2.A.1.40 | AzgA family | 2 | 0 | 0.0 |  |
|  | other MFS | 31 | 13 | 41.9 |  |
| 2.A.3 | APC superfamily | 14 | 4 | 28.6 | amino acids, amines |
| 2.A.3.1 | AAT family | 8 | 2 | 25.0 |  |
|  | other APC | 6 | 2 | 33.3 |  |
| 2.A.4 | CDF family | 1 | 0 | 0.0 | heavy metals (Cd^2+^, Zn^2+^, Co^2+^) |
| 2.A.6 | RND superfamily | 4 | 2 | 50.0 | heavy metals, drugs |
| 2.A.7 | DMT superfamily | 2 | 0 | 0.0 | various |
| 2.A.7.1 | SMR family | 1 | 0 | 0.0 |  |
| 2.A.7.7 | RarD family | 1 | 0 | 0.0 |  |
| 2.A.8 | GntP family | 2 | 1 | 50.0 | D-gluconate, L-idonate |
| 2.A.11 | CitMHS family | 1 | 0 | 0.0 | citrate |
| 2.A.14 | LctP family | 2 | 1 | 50.0 | D-lactate, L-lactate, glycolate |
| 2.A.15 | BCCT family | 4 | 2 | 50.0 | betaine, carnitine, choline |
| 2.A.17 | POT family | 1 | 0 | 0.0 | peptides |
| 2.A.20 | Pit family | 2 | 0 | 0.0 | phosphate, sulfate |
| 2.A.21 | SSS family | 5 | 2 | 40.0 | sugars, amino acids, organo cations |
| 2.A.22 | NSS family | 1 | 1 | 100.0 | neurotransmitters, amino acids, osmolytes, nitrogenous compounds |
| 2.A.23 | DAACS family | 2 | 1 | 50.0 | Krebs cycle dicarboxylates, amino acids |
| 2.A.25 | AGCS family | 1 | 0 | 0.0 | alanine, glycine |
| 2.A.28 | BASS family | 2 | 0 | 0.0 | organic acids |
| 2.A.33 | NhaA family | 2 | 1 | 50.0 | Na+-H+ antiporters |
| 2.A.36 | CPA1 family | 1 | 0 | 0.0 | Na+:H+ antiporters |
| 2.A.38 | Trk family | 1 | 0 | 0.0 | K+:H+ symporters |
| 2.A.39 | NCS1 family | 1 | 0 | 0.0 | nucleobases and related metabolites |
| 2.A.40 | NCS2 family | 2 | 0 | 0.0 | nucleobases |
| 2.A.46 | BenE family | 1 | 0 | 0.0 | benzoate |
| 2.A.47 | DASS family | 1 | 1 | 100.0 | di- and tricarboxylates of the Krebs cycle, amino acids, inorganic sulfate, phosphate |
| 2.A.51 | CHR family | 1 | 0 | 0.0 | chromate, sulfate |
| 2.A.53 | SulP family | 1 | 0 | 0.0 | sulfate |
| 2.A.55 | NramP family | 2 | 1 | 50.0 | metal ions |
| 2.A.59 | ACR3 family | 1 | 0 | 0.0 | arsenite, antimonite |
| 2.A.63 | CPA3 family | 6 | 0 | 0.0 | K+:H+ antiporters, Na+:H+ antiporters |
| 2.A.66 | MOP (MATE) family | 1 | 0 | 0.0 | drugs |
| 2.A.69 | AEC family | 2 | 1 | 50.0 | auxine, malate, malonate |
| 2.A.75 | LysE family | 1 | 0 | 0.0 | amino acids |
| 2.A.76 | RhbT family | 3 | 0 | 0.0 | amino acids |
| 2.A.80 | TTT family | 3 | 0 | 0.0 | citrate |
|  |  |  |  |  |  |
| 3. Primary active transporters | | 197 | 72 | 36.5 |  |
| 3.A.1 | ABC superfamily | 191 | 70 | 36.6 | various |
| 3.A.3 | P-ATPase superfamily | 6 | 2 | 33.3 | cations |
|  |  |  |  |  |  |
| 4. Group translocators | | 1 | 0 | 0.0 |  |
| 4.B.1 | PnuC family | 1 | 0 | 0.0 | nicotinamide ribonucleoside |
|  |  |  |  |  |  |
| 8. Accessory factors involved in transport | | 4 | 0 | 0.0 |  |
| 8.A.5 | Kvß family | 3 | 0 | 0.0 |  |
| 8.A.7 | EI family | 1 | 0 | 0.0 |  |
|  |  |  |  |  |  |
| 9. Incompletely characterized transport systems | | 2 | 0 | 0.0 |  |
| 9.A.19 | MgtE family | 1 | 1 | 100.0 | Mg2+, Co2+ |
| 9.B.74 | PIP family | 1 | 0 | 0.0 | unknown |
|  |  |  |  |  |  |
| Unclassified |  | 10 | 2 | 20.0 |  |
|  |  |  |  |  |  |
| **Total** |  | **356** | **109** | **30.6** |  |

^a^The nomenclature and putative substrates are according to Saier (2000) (Saier MH, Jr. (2000) A Functional-Phylogenetic Classification System for Transmembrane Solute Transporters. Microbiol Mol Biol Rev 64: 354-411). Excluded are PTS systems and transporters involved in protein secretion. Reported are the numbers of genes present for any given category.

^b^No ortholog present in *A. aurescens* TC1, *A. chlorophenolicus* A6 and *Arthrobacter* sp. FB24.
