# Supplementary material for: The Arthrobacter arilaitensis Re117 Genome Sequence Reveals Its Genetic Adaptation to the Surface of Cheese
Source: PLoS One. 2010 Nov 24;5(11):e15489. doi: 10.1371/journal.pone.0015489 (PMC2991359; doi:10.1371/journal.pone.0015489)
Supplement: Table S7 — Predicted transcriptional regulatory proteins of A. arilaitensis Re117. (DOC) [file pone.0015489.s013.doc]

**Table S7** Predicted transcriptional regulatory proteins of *A. arilaitensis* Re117.

|  |  | **Specific for *A. arilaitensis*^a^** | |
| --- | --- | --- | --- |
| **One component regulators** | **Number of proteins** | **Number** | **Percent** |
| Family^b^: |  |  |  |
| AraC | 7 | 4 | 57.1 |
| ArgR | 1 | 0 | 0.0 |
| ArsR | 12 | 3 | 25.0 |
| AsnC | 11 | 1 | 9.1 |
| CarD | 1 | 0 | 0.0 |
| Crp | 2 | 1 | 50.0 |
| DeoR | 5 | 2 | 40.0 |
| DtxR | 3 | 1 | 33.3 |
| FUR | 2 | 0 | 0.0 |
| GntR | 20 | 5 | 25.0 |
| HrcA | 1 | 0 | 0.0 |
| HTH_3 | 12 | 5 | 41.7 |
| IclR | 13 | 5 | 38.5 |
| LacI | 9 | 2 | 22.2 |
| LexA | 1 | 0 | 0.0 |
| LuxR | 1 | 0 | 0.0 |
| LysR | 13 | 3 | 23.1 |
| MarR | 17 | 7 | 41.2 |
| MerR | 9^c^ | 4 | 44.4 |
| PadR | 6^d^ | 3 | 50.0 |
| ROK | 5 | 2 | 40.0 |
| TetR | 24 | 10 | 41.7 |
| WhiB | 2 | 0 | 0.0 |
| Other families or not classified | 22 | 9 | 40.9 |
| Total one component regulators | 199 | 67 | 33.7 |
|  |  |  |  |
| **Two component-systems** |  |  |  |
| Response regulators | 24 | 7 | 29.2 |
| paired | 19 | 4 | 21.1 |
| orphans | 5 | 3 | 60.0 |
| Histidine kinases | 22 | 9 | 40.9 |
| paired | 19 | 6 | 31.6 |
| orphans | 3 | 3 | 100.0 |
| Hybrids | 0 | 0 | / |
| Total two-component proteins | 46 | 16 | 34.8 |
|  |  |  |  |
| **Sigma factors** | 7 | 2 | 28.6 |
| **Anti-sigma factors** | 1 | 1 | 100 |
|  |  |  |  |
| Total | 253 | 86 | 34.0 |
| % of CDS | 7.4 |  |  |

^a^No ortholog in *A. aurescens* TC1, *A. chlorophenolicus* A6 and *Arthrobacter* sp. FB24.

^b^The protein families were named according to designations by the pfam database. Only proteins with either a Pfam or COG hit were counted.

^c^Including one CDS on plasmid pRE117-1.

^d^Including one CDS on plasmid pRE117-2.
