# Supplementary material for: The Arthrobacter arilaitensis Re117 Genome Sequence Reveals Its Genetic Adaptation to the Surface of Cheese
Source: PLoS One. 2010 Nov 24;5(11):e15489. doi: 10.1371/journal.pone.0015489 (PMC2991359; doi:10.1371/journal.pone.0015489)
Supplement: Table S8 — Catabolism of amino acids by A. arilaitensis Re117. (DOC) [file pone.0015489.s014.doc]

**Table S8** Catabolism of amino acids by *A. arilaitensis* Re117.

| Amino acid | Catabolic pathway predicted from genome analysis | Use of amino acid for growth on Biotype 100 strips |
| --- | --- | --- |
| Lysine | Absence lysine monooxygenase (EC 1.13.12.2), L-lysine 6-transaminase (EC 2.6.1.36), L-lysine oxidase (EC 1.4.3.14), lysine 6-dehydrogenase (EC 1.4.1.18), lysine decarboxylase (EC 4.1.1.18) and lysine racemase (EC 5.1.1.5) pathways | NA^a^ |
| Proline | Presence of catabolic pathway: conversion of proline to glutamate by the bifunctional proline dehydrogenase/pyrroline-5-carboxylate dehydrogenase (EC 1.5.99.8 and EC 1.5.1.12) | Yes |
| Glutamate | Presence of catabolic pathway: conversion of glutamate to α-ketoglutarate by the glutamate dehydrogenase (EC 1.4.1.2) | Yes |
| Tyrosine | Incomplete catabolic pathway: no candidates for the 4-hydroxyphenylpyruvate oxidase (EC 1.2.3.13) and the 4-hydroxyphenylacetate 3-monooxygenase (EC 1.14.13.3), wich are involved in the catabolim via homoprotocatechuate | Yes |
| Histidine | Presence of catabolic pathway: conversion of histidine to glutamate by action of the histidase (EC 4.3.1.3), urocanase (EC 4.2.1.49), imidazolonepropionase (EC 3.5.2.7) and formimidoylglutamase (EC 3.5.3.8) | No |
| Tryptophan | Absence of the tryptophanase (EC 4.1.99.1), L-tryptophan transaminase (EC 2.6.1.27) and L-tryptophan decarboxylase (EC 4.1.1.28) pathways; incomplete tryptophan dioxygenase (EC 1.13.11.11) pathway | No |
| Phenylalanine | Absence of the phenylalanine 4-monooxygenase (EC 1.14.16.1) pathway | NA |
| Arginine | Absence of arginine deiminase (EC 3.5.3.6), arginine N-succinyltransferase (EC 2.3.1.109), arginine decarboxylase (EC 4.1.1.19), arginine:pyruvate transaminase (EC 2.6.1.84), arginine oxidase (EC 1.4.3.2), arginine 2-monnooxygenase (EC 1.13.12.1), arginine-α-ketoglutarate transaminase and arginase (EC 3.5.3.1) pathways | NA |
| Isoleucine | Presence of catabolic pathway: conversion of isoleucine to propionyl-CoA and acetyl-CoA | NA |
| Leucine | Incomplete catabolic pathway: no candidates for the methylcrotonoyl-CoA carboxylase (EC 6.4.1.4), the methylglutaconyl-CoA hydratase (EC 4.2.1.18) and the hydroxymethylglutaryl-CoA lyase (EC 4.1.3.4) | NA |
| Valine | Incomplete catabolic pathway: no candidates for the 3-hydroxyisobutyryl-CoA hydrolase (EC 3.1.2.4) | NA |
| Glycine | Presence of catabolic pathway: glycine cleavage system (EC 1.4.4.2, EC 2.1.2.10 and EC 1.8.1.4) | NA |
| Threonine | Presence of the threonine aldolase (EC 4.1.2.5), threonine dehydrogenase (EC 1.1.1.103), and threonine dehydratase (EC 4.3.1.19) pathways | NA |
| Serine | Presence of catabolic pathway: conversion of serine to pyruvate by the serine deaminase (EC 4.3.1.17) | Yes |
| Alanine | Presence of the alanine transferase (EC 2.6.1.2) pathway  Absence of the alanine dehydrogenase (EC 1.4.1.1) pathway | Yes |
| Aspartate | Presence of catabolic pathway: conversion of aspartate to oxaloacetate by the reversible aspartate aminotransferase (EC 2.6.1.1) | Yes |
| Methionine | Absence of the L-methionine-γ-lyase (EC 4.4.1.11) pathway | NA |
| Cysteine | Absence of the cystathionine γ-lyase (EC 4.4.1.1) pathway | NA |
| 4-Aminobutyrate | Presence of catabolic pathway: conversion of 4-aminobutyrate to succinate by action of the 4-aminobutyrate transaminase (EC 2.6.1.19) and the succinate-semialdehyde dehydrogenase (EC 1.2.1.16) | No |

^a^NA: not available.
