# Supplementary material for: The Arthrobacter arilaitensis Re117 Genome Sequence Reveals Its Genetic Adaptation to the Surface of Cheese
Source: PLoS One. 2010 Nov 24;5(11):e15489. doi: 10.1371/journal.pone.0015489 (PMC2991359; doi:10.1371/journal.pone.0015489)
Supplement: Table S9 — Utilization of various carbon sources by Arthrobacter strains growing aerobically on Biotype 100 strips. (DOC) [file pone.0015489.s015.doc]

**Table S9** Utilization of various carbon sources by *Arthrobacter* strains growing aerobically on Biotype 100 strips.

| Substrate | *A. arilaitensis* Re117 | *A. aurescens* TC1 | *Arthrobacter* sp. FB24 | *A. chlorophenolicus* A6 |
| --- | --- | --- | --- | --- |
|  |  |  |  |  |
| D-Glucose | + | + | + | + |
| D-Fructose | - | + | + | + |
| D-Galactose | + | + | + | + |
| D-Trehalose | + | + | + | + |
| D-Mannose | - | + | + | + |
| L-Sorbose | - | - | - | - |
| D-Melibiose | - | + | + | + |
| Sucrose | + | + | + | + |
| D-Raffinose | - | + | + | + |
| Maltotriose | + | + | + | + |
| Maltose | + | + | + | + |
| Lactose | + | - | + | + |
| Lactulose | - | + | + | + |
| 1-O-Methyl-β-galactoside | - | - | + | + |
| 1-O-Methyl-α-galactoside | - | - | + | + |
| D-Cellobiose | + | + | + | + |
| Gentiobiose | - | + | + | + |
| 1-O-Methyl-β-D-glucoside | - | + | + | + |
| Esculin | + | + | + | + |
| D-Ribose | + | - | + | - |
| L-Arabinose | + | + | + | + |
| D-Xylose | + | + | + | + |
| Palatinose | - | + | + | + |
| L-Rhamnose | - | - | + | - |
| L-Fucose | - | - | - | - |
| D-Melezitose | - | + | + | + |
| D-Arabitol | + | - | - | - |
| L-Arabitol | - | - | + | - |
| Xylitol | - | + | - | + |
| Dulcitol | - | - | - | - |
| D-Tagatose | - | - | - | - |
| Glycerol | + | + | + | + |
| myo-Inositol | - | + | + | + |
| D-Mannitol | - | + | + | + |
| Maltitol | - | + | + | + |
| D-Turanose | + | + | + | + |
| D-Sorbitol | - | - | - | + |
| Adonitol | - | - | - | - |
| Hydroxyquinoline-β-glucuronide | - | - | - | - |
| D-Lyxose | - | - | - | - |
| i-Erythritol | - | - | - | - |
| 1-O-Methyl-α-D-glucoside | - | - | - | - |
| 3-O-Methyl-D-glucose | - | - | - | - |
| D-Saccharate | - | - | - | + |
| Mucate | - | - | - | - |
| L-Tartrate | - | - | - | + |
| D-Tartrate | - | - | - | - |
| meso-Tartrate | - | - | - | - |
| D-Malate | - | - | + | + |
| L-Malate | - | + | + | + |
| cis-Aconitate | - | + | + | + |
| trans-Aconitate | - | + | + | + |
| Tricarballylate | - | - | - | - |
| Citrate | - | + | + | + |
| D-Glucuronate | - | + | + | + |
| D-Galacturonate | - | - | - | - |
| 2-Ketogluconate | - | + | + | + |
| 5-Ketogluconate | + | - | + | - |
| Tryptophan | - | - | - | - |
| N-Acetyl-D-glucosamine | - | - | + | - |
| D-Gluconate | + | + | + | + |
| Phenylacetate | - | + | + | + |
| Protocatechuate | + | + | + | + |
| 4-Hydroxybenzoate | + | + | + | + |
| Quinate | - | + | + | + |
| Gentisate | - | + | + | + |
| 3-Hydroxybenzoate | - | + | + | + |
| Benzoate | - | - | - | - |
| 3-Phenylpropionate | - | + | + | + |
| m-Coumarate | - | + | + | + |
| Trigonelline | - | - | - | - |
| Betaine | - | + | - | - |
| Putrescine | + | + | + | + |
| 4-Aminobutyrate | - | + | + | + |
| Histamine | - | + | - | - |
| DL-Lactate | + | + | + | - |
| Caprate | - | - | - | - |
| Caprylate | - | + | + | + |
| L-histidine | - | - | + | - |
| Succinate | - | + | + | + |
| Fumarate | - | + | + | + |
| Glutarate | + | - | - | - |
| DL-Glycerate | + | - | - | - |
| 5-Aminovalerate | - | - | - | - |
| Ethanolamine | - | - | - | - |
| Tryptamine | - | - | - | - |
| D-Glucosamine | - | - | + | + |
| Itaconate | - | - | - | - |
| 3-Hydroxybutyrate | + | + | + | + |
| L-Aspartate | + | + | + | + |
| L-Glutamate | + | + | + | + |
| L-Proline | + | + | + | + |
| D-Alanine | - | + | + | + |
| L-Alanine | + | + | + | + |
| L-Serine | + | + | - | + |
| Malonate | + | - | + | + |
| Propionate | - | + | + | - |
| L-Tyrosine | + | + | + | + |
| 2-Ketoglutarate | - | - | + | + |
| D-Galactono-γ-lactone^a^ | + | - | - | - |
| **Number of carbon sources utilized:** | **32** | **57** | **66** | **63** |

^a^ D-Galactono-γ-lactone was tested separately, as described in Materials and Methods.
