# Supplementary material for: The Arthrobacter arilaitensis Re117 Genome Sequence Reveals Its Genetic Adaptation to the Surface of Cheese
Source: PLoS One. 2010 Nov 24;5(11):e15489. doi: 10.1371/journal.pone.0015489 (PMC2991359; doi:10.1371/journal.pone.0015489)
Supplement: Table S10 — Occurence of COG hits related to sugar ABC-type transport systems. (DOC) [file pone.0015489.s016.doc]

**Table S10** Occurence of COG hits related to sugar ABC-type transport systems.

| COG no | Name | *A. arilaitensi*s Re117 | *A. aurescens* TC1 | *A. chlorophenolicus* A6 | *Arthrobacter* sp. FB24 |
| --- | --- | --- | --- | --- | --- |
|  |  |  |  |  |  |
| 0395 | ABC-type sugar transport system, permease component | 5 | 26 | 20 | 24 |
| 1129 | ABC-type sugar transport system, ATPase component | 2 | 7 | 4 | 8 |
| 1172 | Ribose/xylose/arabinose/galactoside ABC-type transport systems, permease components | 1 | 4 | 2 | 5 |
| 1175 | ABC-type sugar transport systems, permease components | 5 | 25 | 19 | 22 |
| 1653 | ABC-type sugar transport system, periplasmic component | 4 | 25 | 24 | 26 |
| 1682 | ABC-type polysaccharide/polyol phosphate export systems, permease component | 1 | 3 | 2 | 2 |
| 1869 | ABC-type ribose transport system, auxiliary component | 1 | 0 | 0 | 0 |
| 1879 | ABC-type sugar transport system, periplasmic component | 1 | 4 | 3 | 6 |
| 2182 | Maltose-binding periplasmic proteins/domains | 1 | 2 | 0 | 2 |
| 3822 | ABC-type sugar transport system, auxiliary component | 0 | 0 | 0 | 0 |
| 3833 | ABC-type maltose transport systems, permease component | 1 | 1 | 0 | 1 |
| 3839 | ABC-type sugar transport systems, ATPase components | 1 | 1 | 1 | 1 |
| 4158 | Predicted ABC-type sugar transport system, permease component | 0 | 0 | 1 | 1 |
| 4209 | ABC-type polysaccharide transport system, permease component | 0 | 2 | 1 | 2 |
| 4211 | ABC-type glucose/galactose transport system, permease component | 0 | 0 | 0 | 0 |
| 4213 | ABC-type xylose transport system, periplasmic component | 1 | 2 | 1 | 1 |
| 4214 | ABC-type xylose transport system, permease component | 1 | 2 | 1 | 1 |
| **total numbers:** | | **25** | **104** | **79** | **102** |
