# Supplementary material for: The Arthrobacter arilaitensis Re117 Genome Sequence Reveals Its Genetic Adaptation to the Surface of Cheese
Source: PLoS One. 2010 Nov 24;5(11):e15489. doi: 10.1371/journal.pone.0015489 (PMC2991359; doi:10.1371/journal.pone.0015489)
Supplement: Table S11 — Genes in the A. arilaitensis Re117 genome with putative function in protein degradation. (DOC) [file pone.0015489.s017.doc]

**Table S11** Genes in the *A. arilaitensis* Re117 genome with putative function in protein degradation.^a^

| **Locus tag**  **AARI_** | **Gene symbol** | **EC number** | **Signal peptide^b^** | **Predicted protein product** |
| --- | --- | --- | --- | --- |
|  |  |  |  |  |
| Chromosome | |  |  |  |
| 00130 |  |  |  | rhomboid family protein |
| 00760 | *pcp* | 3.4.19.3 |  | pyroglutamyl-peptidase I |
| 00840 | *ftsH* | 3.4.24.- |  | putative cell division protein FtsH |
| 00980 |  | 3.4.24.- |  | putative metalloproteinase |
| 02060 |  | 3.4.24.- |  | putative membrane-bound M23 family peptidase |
| 03530 |  | 3.4.21.- | + | secreted subtilase family protease |
| 03820 |  | 3.4.-.- | + | putative secreted M23 family peptidase |
| 05120 |  | 3.4.21.- |  | prolyl oligopeptidase family protein |
| 05180 |  | 3.4.-.- | + | putative secreted peptidase |
| 05620 |  | 3.4.24.- |  | putative M23 family peptidase |
| 07280 |  |  |  | putative metal-dependent amidase/aminoacylase/carboxypeptidase |
| 07430 |  | 3.4.21.- | + | membrane-associated subtilase family protease |
| 07840 |  |  |  | putative transglutaminase-like protease |
| 09630 |  | 3.4.24.- | + | putative secreted M23 family peptidase |
| 09970 |  | 3.4.24.- |  | zinc metallopeptidase |
| 10740 |  | 3.4.24.- |  | putative M16 family peptidase |
| 11400 |  | 3.4.11.- |  | M1 family aminopeptidase |
| 12200 |  | 3.4.24.- | + | putative secreted M23 family peptidase |
| 12910 | *clpS* |  |  | ATP-dependent Clp protease adaptor protein ClpS |
| 16040 | *pepN* | 3.4.11.2 |  | membrane alanyl aminopeptidase |
| 16180 |  | 3.4.11.5 |  | putative prolyl aminopeptidase |
| 16260 |  | 3.4.-.- |  | putative zinc metallopeptidase |
| 16340 |  |  |  | zinc metallopeptidase |
| 16360 |  |  |  | putative ATP-dependent 26S proteasome regulatory subunit |
| 16370 |  |  |  | putative proteasome component |
| 16390 |  |  |  | putative proteasome component |
| 16730 |  | 3.4.-.- |  | putative zinc metallopeptidase |
| 16830 |  | 3.4.24.- |  | putative metalloprotease |
| 17550 | *dcp* | 3.4.15.5 |  | peptidyl-dipeptidase |
| 18500 | *clpX* |  |  | ATP-dependent Clp protease ATP-binding subunit ClpX |
| 18510 | *clpP* | 3.4.21.92 |  | ATP-dependent Clp protease proteolytic subunit ClpP |
| 18520 | *clpP* | 3.4.21.92 |  | ATP-dependent Clp protease proteolytic subunit ClpP |
| 18560 | *pepN* | 3.4.11.2 |  | membrane alanyl aminopeptidase |
| 19220 |  | 3.4.11.5 |  | putative prolyl aminopeptidase |
| 20260 | *pepA* | 3.4.11.1 |  | leucyl aminopeptidase |
| 21260 |  | 3.4.21.- |  | putative serine protease |
| 21580 | *pepP* | 3.4.11.9 |  | Xaa-Pro aminopeptidase |
| 23020 | *gcp* | 3.4.24.57 |  | probable O-sialoglycoprotein endopeptidase |
| 23040 |  |  |  | putative glycoprotease |
| 24060 |  | 3.4.-.- |  | M18 family aminopeptidase |
| 24070 | *htpX* | 3.4.24.- |  | protease HtpX homolog |
| 24380 | *ptrB* | 3.4.21.83 |  | putative oligopeptidase B |
| 25030 |  | 3.4.-.- |  | putative membrane-associated serine protease |
| 26640 |  | 3.4.21.- |  | prolyl oligopeptidase family protein |
| 27010 |  |  |  | putative metal-dependent amidase/aminoacylase/carboxypeptidase |
| 27930 |  | 3.4.21.- | + | secreted subtilase family protease |
| 28640 |  | 3.4.21.- |  | prolyl oligopeptidase family protein |
| 30150 |  | 3.4.24.- |  | putative M23 family peptidase |
| 31870 |  | 3.4.-.- |  | PfpI family intracellular protease |
| 32140 |  |  |  | putative carboxypeptidase |
|  |  |  |  |  |
| Plasmid pRE117-1 | |  |  |  |
| AARI_pI00350 |  | 3.4.24.- | + | putative secreted M23 family peptidase |

^a^ The *A. arilaitensis* genes having no ortholog in *A. aurescens* TC1, *A. chlorophenolicus* A6 and *Arthrobacter* sp. FB24 are underlined.

^b^ Putative signal sequences were predicted using SignalP 3.0 HMM (signal peptidase I cleavage site).
