# Supplementary material for: The Arthrobacter arilaitensis Re117 Genome Sequence Reveals Its Genetic Adaptation to the Surface of Cheese
Source: PLoS One. 2010 Nov 24;5(11):e15489. doi: 10.1371/journal.pone.0015489 (PMC2991359; doi:10.1371/journal.pone.0015489)
Supplement: Table S12 — Genes assigned to glycine betaine and related osmolyte transporters in the A. arilaitensis genome. (DOC) [file pone.0015489.s018.doc]

**Table S12** Genes assigned to glycine betaine and related osmolyte transporters in the *A. arilaitensis* genome.^a^

|  |  | *A. arilaitensi*s Re117 | | *A. aurescens* TC1 | | *Arthrobacter* sp. FB24 | | *A. chlorophenolicus* A6 | |
| --- | --- | --- | --- | --- | --- | --- | --- | --- | --- |
| Function | COG no | number | Locus tag AARI_ | number | Locus tag  Aaur_ | number | Locus tag  Arth_ | number | Locus tag  Achl_ |
| ABC-type proline glycine betaine transport system, ATPase component | 1125, 4175 | 3 | 10830, 15200, 28120 | 2 | 0645, 2817 | 1 | 2842 | 1 | 2557 |
| ABC-type proline glycine betaine transport system, permease component | 1174, 4176 | 5 | 10840, 10850, 15190, 28110, 28130 | 4 | 0644, 0646, 2815, 2816 | 2 | 2840, 2841 | 2 | 2555, 2556 |
| ABC-type proline glycine betaine transport system, substrate binding component | 1732, 2113 | 3 | 10860, 15180, 28100, | 2 | 0647, 2814 | 1 | 2839 | 1 | 2554 |
| Choline-glycine betaine transporter | 1292 | 4 | 02520, 07250, 27130, 27240 | 1 | 0477 | 2 | 3716, 3745 | 4 | 0079, 3341, 3457, 3496 |
| **Total numbers:** |  | **15** |  | **9** |  | **6** |  | **8** |  |
|  |  |  |  |  |  |  |  |  |  |

^a^ The *A. arilaitensis* genes with no ortholog in any of the three environmental *Arthrobacter* strains are underlined.
