# Supplementary material for: The Arthrobacter arilaitensis Re117 Genome Sequence Reveals Its Genetic Adaptation to the Surface of Cheese
Source: PLoS One. 2010 Nov 24;5(11):e15489. doi: 10.1371/journal.pone.0015489 (PMC2991359; doi:10.1371/journal.pone.0015489)
Supplement: Table S13 — Predicted lipase and esterase genes in A. arilaitensis Re117. (DOC) [file pone.0015489.s019.doc]

**Table S13** Predicted lipase and esterase genes in *A. arilaitensis* Re117.^a^

| **Locus tag**  **AARI_** | **Gene symbol** | **EC number** | **Signal peptide^b^** | **Predicted protein product** |
| --- | --- | --- | --- | --- |
|  | |  |  |  |
| 00520 | glpQ | 3.1.4.46 |  | putative glycerophosphodiester phosphodiesterase |
| 01720 | glpQ | 3.1.4.46 |  | putative glycerophosphodiester phosphodiesterase |
| 04500 |  | 3.1.1.3 | + | secreted triacylglycerol lipase |
| 04510 |  | 3.1.-.- |  | phospholipase/carboxylesterase family protein |
| 04810 | glpQ | 3.1.4.46 |  | putative glycerophosphodiester phosphodiesterase |
| 09200 |  | 3.1.1.- |  | GDSL-like esterase/lipase |
| 09240 |  | 3.1.-.- |  | phospholipase/carboxylesterase family protein |
| 11770 |  | 3.1.1.- |  | GDSL-like esterase/lipase |
| 12030 |  | 3.1.1.- |  | putative esterase/lipase |
| 12200 |  | 3.4.24.- | + | putative secreted M23 family peptidase |
| 12340 |  | 3.1.1.- |  | putative esterase/lipase |
| 13420 |  | 3.1.1.- |  | putative esterase/lipase |
| 17050 |  | 3.1.1.- |  | putative esterase/lipase |
| 19870 |  | 3.1.1.- | + | putative esterase/lipase |
| 26230 |  | 3.1.1.- |  | GDSL-like esterase/lipase |
| 29330 |  | 3.1.1.5 |  | possible lysophospholipase |
| 30760 |  | 3.1.1.- |  | GDSL-like esterase/lipase |
| 32100 |  | 3.1.4.46 |  | putative periplasmic glycerophosphoryl diester phosphodiesterase |

^a^ The *A. arilaitensis* genes having no ortholog in *A. aurescens* TC1, *A. chlorophenolicus* A6 and *Arthrobacter* sp. FB24 are underlined.

^b^ Putative signal sequences were predicted using SignalP 3.0 HMM (signal peptidase I cleavage site).
