# Supplementary material for: The Arthrobacter arilaitensis Re117 Genome Sequence Reveals Its Genetic Adaptation to the Surface of Cheese
Source: PLoS One. 2010 Nov 24;5(11):e15489. doi: 10.1371/journal.pone.0015489 (PMC2991359; doi:10.1371/journal.pone.0015489)
Supplement: Table S14 — Predicted acyl-CoA dehydrogenase and fatty acid--CoA ligase genes in A. arilaitensis Re117. (DOC) [file pone.0015489.s020.doc]

**Table S14** Predicted acyl-CoA dehydrogenase and fatty acid--CoA ligase genes in *A. arilaitensis* Re117.^a^

|  | Orthologs in: | | |
| --- | --- | --- | --- |
| Locus tag AARI_ | *A. aurescens* TC1  Locus tag AAur_ | *Arthrobacter* sp*.* FB24  Locus tag Arth_ | *A. chlorophenolicus* A6 Locus tag Achl_ |
| Acyl-CoA dehydrogenase: |  |  |  |
| 02570 | / | / | / |
| 02680 | / | / | / |
| 05890 | 3029 | 3055 | 2757 |
| 09950 | 1530 | 1387 | 1405 |
| 15780 | / | / | / |
| 16270 | 2145 | 2163 | 1906 |
| 22150 | 0963 | 3822 | 1382 |
| 26490 | / | 1365 | 1382 |
| 28290 | / | / | / |
| 32740 | / | / | / |
| 32750 | pTC20121 | / | / |
|  |  |  |  |
| Fatty acid--CoA ligase: |  |  |  |
| 07800 | 1328 | 1204 | 1274 |
| 10560 | 1999 | / | 0447 |
| 12010 | 1687 | 1547 | 1548 |
| 14920 | / | / | / |
| 14930 | 0509 | 0433 | / |
| 18740 | 2110 | 2109 | 1851 |
| 22140 | 0966 | 3819 | / |
| 28430 | / | / | / |
| 29000 | / | / | / |
| 33060 | 3043 | 3066 | 2768 |
| 33910 | / | / | / |

^a^ The *A. arilaitensis* genes having no ortholog in any of the three environmental *Arthrobacter* strains are underlined.
