# Supplementary material for: The Arthrobacter arilaitensis Re117 Genome Sequence Reveals Its Genetic Adaptation to the Surface of Cheese
Source: PLoS One. 2010 Nov 24;5(11):e15489. doi: 10.1371/journal.pone.0015489 (PMC2991359; doi:10.1371/journal.pone.0015489)
Supplement: Table S15 — Distribution of ABC families in Arthrobacter arilaitensis Re117. (DOC) [file pone.0015489.s021.doc]

**Table S15** Distribution of ABC families in *Arthrobacter arilaitensis* Re117.

|  |  | Specific for  *A. arilaitensis*^b^ | |  |
| --- | --- | --- | --- | --- |
| Family | Number*^a^* | Number | Percent | Putative substrates |
|  |  |  |  |  |
| OTCN | 18 | 6 | 33.3 | osmoprotectants, taurine, cyanate and nitrate |
| NO | 3 | 0 | 0.0 | unclassified systems |
| OPN | 34 | 10 | 29.4 | oligopeptides and nickel |
| DRI | 9 | 3 | 33.3 | drug resistance, bacteriocin and lantibiotic immunity |
| DRA | 4 | 1 | 25.0 | drug and antibiotic resistance |
| DPL | 12 | 7 | 58.3 | drugs, peptides, lipids |
| MOI | 13 | 7 | 53.8 | mineral and organic |
| o228 | 7 | 3 | 42.9 | release of lipoproteins and drug resistance |
| CBY | 6 | 2 | 33.3 | cobalt uptake, unknown |
| ISVH | 28 | 18 | 64.3 | iron siderophores, vitamin B12 and hemin |
| MET | 10 | 2 | 20.0 | metals |
| MOS | 11 | 4 | 36.4 | monosaccharides |
| OSP | 15 | 1 | 6.7 | oligosaccharides and polyols |
| PAO | 12 | 1 | 8.3 | polar amino acid and opines |
| DLM | 6 | 3 | 50.0 | D- L-methionine and derivatives |
| CLS | 2 | 2 | 100.0 | capsular polysaccharide, lipopolysaccharide, teichoic acids |
| YLU | 1 | 0 | 0 | unclassified systems |
| **Total :** | **191** | **70** | **36.6** |  |

^a^Reported are the number of genes present for any given category.

^b^No ortholog present in *A. aurescens* TC1, *A. chlorophenolicus* A6 and *Arthrobacter* sp. FB24.
